# Ythdf2-mediated m^6^A mRNA clearance modulates neural development in mice

**DOI:** 10.1186/s13059-018-1436-y

**Published:** 2018-05-31

**Authors:** Miaomiao Li, Xu Zhao, Wei Wang, Hailing Shi, Qingfei Pan, Zhike Lu, Sonia Peña Perez, Rajikala Suganthan, Chuan He, Magnar Bjørås, Arne Klungland

**Affiliations:** 1 Department of Microbiology, Oslo University Hospital, Rikshospitalet, NO-0027 Oslo, Norway; 2 Department of Molecular Medicine, Institute of Basic Medical Sciences, University of Oslo, NO-0317 Oslo, Norway; 3 Department of Clinical and Molecular Medicine, Norwegian University of Science and Technology (NTNU), Trondheim, Norway; 4 Department of Chemistry, Institute for Biophysical Dynamics, The University of Chicago, 929 East 57th Street, Chicago, IL 60637 USA; 5 Howard Hughes Medical Institute, The University of Chicago, 929 East 57th Street, Chicago, IL 60637 USA; 6 Department of Computational Biology, St. Jude Children’s Hospital, Memphis, TN 38105 USA

**Keywords:** Ythdf2, *N*^*6*^-methyladenosine (m^6^A), Neural development, Neurogenesis, mRNA clearance

## Abstract

**Background:**

*N*^*6*^-methyladenosine (m^6^A) modification in mRNAs was recently shown to be dynamically regulated, indicating a pivotal role in multiple developmental processes. Most recently, it was shown that the Mettl3-Mettl14 writer complex of this mark is required for the temporal control of cortical neurogenesis. The m^6^A reader protein Ythdf2 promotes mRNA degradation by recognizing m^6^A and recruiting the mRNA decay machinery.

**Results:**

We show that the conditional depletion of the m^6^A reader protein Ythdf2 in mice causes lethality at late embryonic developmental stages, with embryos characterized by compromised neural development. We demonstrate that neural stem/progenitor cell (NSPC) self-renewal and spatiotemporal generation of neurons and other cell types are severely impacted by the loss of Ythdf2 in embryonic neocortex. Combining in vivo and in vitro assays, we show that the proliferation and differentiation capabilities of NSPCs decrease significantly in *Ythdf2*^*−/−*^ embryos. The *Ythdf2*^*−/−*^ neurons are unable to produce normally functioning neurites, leading to failure in recovery upon reactive oxygen species stimulation. Consistently, expression of genes enriched in neural development pathways is significantly disturbed. Detailed analysis of the m^6^A-methylomes of *Ythdf2*^*−/−*^ NSPCs identifies that the JAK-STAT cascade inhibitory genes contribute to neuroprotection and neurite outgrowths show increased expression and m^6^A enrichment. In agreement with the function of Ythdf2, delayed degradation of neuron differentiation-related m^6^A-containing mRNAs is seen in *Ythdf2*^*−/−*^ NSPCs.

**Conclusions:**

We show that the m^6^A reader protein Ythdf2 modulates neural development by promoting m^6^A-dependent degradation of neural development-related mRNA targets.

**Electronic supplementary material:**

The online version of this article (10.1186/s13059-018-1436-y) contains supplementary material, which is available to authorized users.

## Background

Over the past decade, more than 100 post-transcriptionally modified ribonucleotides have been identified in various types of RNA [1]. Much more recently, epitranscriptomic [2] regulation at the RNA level via reversible RNA methylation has been revealed, beginning from 2011 with the discovery of the reversible potential of *N*^*6*^-methyl-adenosine (m^6^A) in mRNA [3]. As a post-transcriptional epitranscriptomic modification, m^6^A is one of the most abundant modifications in mRNA in eukaryotes [4]. It can be written by the methyltransferase complex (Mettl3, Mettl14, Wtap, and Kiaa1429) [5], erased by demethylases (Fto and Alkbh5) [3, 6], and read by the binding proteins (Ythdf1–3, Ythdc1–2, and Hnrnp family proteins) [7–10].

The reversible/dynamic nature of m^6^A in mRNA and the ability to map this modification transcriptome-wide have led to a tremendous increase in the interest and understanding of the multiple biological roles of the dynamic m^6^A modification [10, 11]. One of the evolutionarily conserved roles of the m^6^A modification is the regulation of meiosis and fertility. This was shown early for the writers of m^6^A in model organisms [12] and also for the mammalian m^6^A eraser Alkbh5 [6] and the m^6^A reader protein Ythdf2 [13]. The depletion of the m^6^A eraser Fto in mammalian cells causes defects in energy homeostasis and adipocyte differentiation [14]. It is worth mentioning that a loss-of-function mutation in the Fto gene causes growth retardation and multiple malformations in humans [15]. The writer Mettl3 is crucial for maintaining mouse stem cell pluripotency, regulating the reprogramming of somatic cells and the circadian rhythm, and targeting of the gene in mouse causes early embryonic lethality [16–20]. The most recent studies in hematopoietic stem/progenitor cells have uncovered the crucial role of Mettl3 in determining cell fates during vertebrate embryogenesis [21, 22]. The m^6^A reader proteins Ythdf1–3 share a set of common mRNA targets and spatiotemporal interplay with each other cooperatively control translation and decay of these common targets in the cytosol [23]. The m^6^A readers Ythdc1 and Hnrnpa2b1 regulate splicing and processing of their mRNA targets [8, 24], while Ythdc2 affects translation efficiency as well as stability of target mRNAs [9].

Recently, mutant models of the mammalian m^6^A readers reveal interesting phenotypes, which again include spermatogenesis [9] and oocyte competence [25]. Moreover, Ythdf2-dependent, m^6^A-modified mRNA clearance was shown to impact the highly regulated maternal-to-zygotic transition (MZT) in zebrafish [7, 13]. In *Drosophila*, m^6^A writer (Ime4, dMettl14) and reader (Yt521-b) mutants exhibit flight defects and poor locomotion due to impaired neuronal functions [26]. Most recently, the m^6^A writer Mettl14 was shown to be required for the temporal control of mammalian cortical neurogenesis [27]. These findings strongly suggest the potential role of m^6^A modification during nervous system development, which might be conserved across species. Many histone and DNA encoded epigenetic mechanisms are uncovered to be conserved in this process. Thus, addressing the role of m^6^A methylation in mRNA will be an exciting new field to explore and will shed new light on neural development.

The m^6^A reader Ythdf2 is essential for oocyte competence and mutation of it causes female infertility [25]. Here we describe the early brain developmental failure of mice lacking Ythdf2 due to failure to regulate neural stem/progenitor cell (NSPC) proliferation and differentiation. During embryonic development, apical progenitor cells in the ventricular zone (VZ) serve as primitive neural stem cells that give rise to both the neuronal and glial lineages directly or produce secondary progenitors, termed the basal progenitor, in the subventricular zone (SVZ) in a precisely regulated spatiotemporal order [28]. In this study, *Ythdf2* knockout embryos displayed delayed cortical neurogenesis. In vivo and in vitro experiments proved that *Ythdf2*-deficient NSPCs display decreased proliferation rates. Furthermore, *Ythdf2*-deficient NSPCs could naturally differentiate to neurons but not glial cells in vitro. However, the properties of differentiated neurons were influenced, seen as less neurite outgrowth and shorter neurites. Removal of *Ythdf2* increased the sensitivity of neurons to reactive oxygen species (ROS) stress and decreased their recovery capability. RNA-seq combined with m^6^A-seq uncovered that the m^6^A-modified mRNAs involved in negative regulation of neural development were up-regulated in *Ythdf2*-deficient NSPCs, in agreement with the function of Ythdf2. The m^6^A-modified mRNA targets, recognized by the Ythdf2 protein in the wild type, were characterized by delayed degradation in *Ythdf2* knockout embryos. Taken together, our findings reveal the critical functions of m^6^A modification and its binding protein Ythdf2 in neural development.

## Results and discussion

### *Ythdf2*^*−/−*^ targeted mice are embryonic lethal

**Fig. 1:**
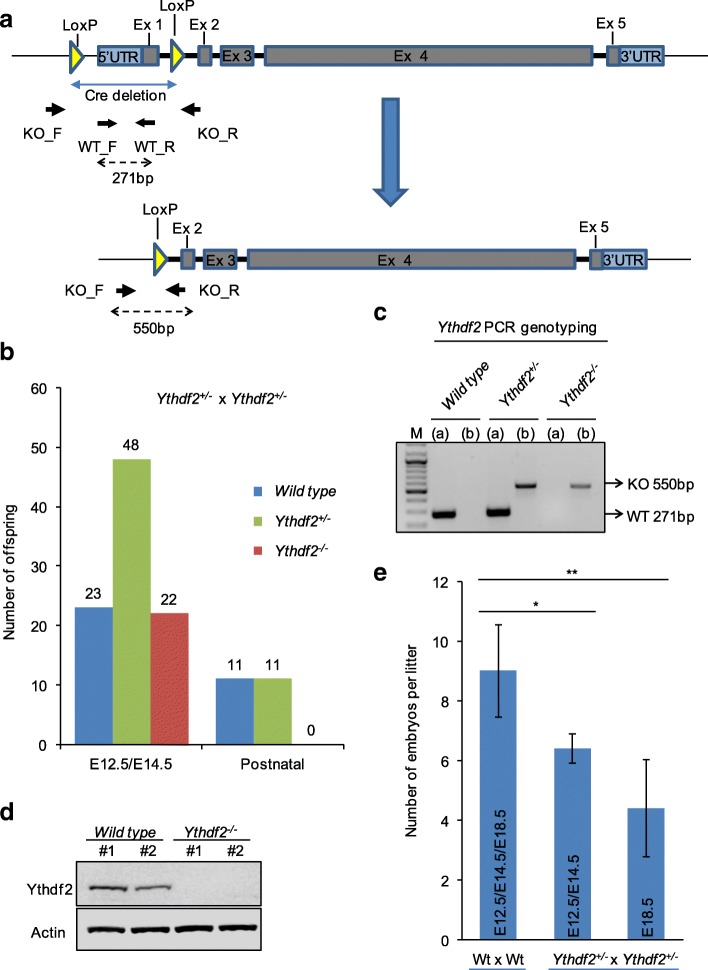
*Ythdf2*^*−/−*^ mice are embryonic lethal. **a** The gene-targeting strategy to disrupt the *Ythdf2* gene in mouse. Conditional *Ythdf2* gene-targeted mouse contains LoxP sites flanking the 5′ UTR and exon 1 of the endogenous *Ythdf2* locus. *WT_F* wild-type forward primer, *WT_R* wild-type reverse primer, *KO_F Ythdf2*^*−/−*^ forward primer, *WT_R Ythdf2*^*−/−*^ reverse primer, *Ex* exon. **b** Numbers of offspring from heterozygous *Ythdf2*^+/−^ intercrosses. The number and genotype of embryos at E12.5/E14.5 and postnatal are indicated. **c** PCR analysis of embryo tail DNA showing a 271-bp wild-type band (*WT*) and a 550-bp targeted band (*KO*) with primers displayed in **a**. **d** Western blot analysis of the Ythdf2 expression in wild-type and *Ythdf2*^*−/−*^ embryos. Two samples for each genotype. Actin was used as loading control. **e** Numbers of embryos per litter at E12.5/E14.5 and E18.5 from wild-type or heterozygous intercrosses. *Error bars* represent mean ± standard deviation, *n* = 7 litters. *P < 0.05, **P < 0.01, ***P < 0.001, Student’s *t*-test

In order to study the biological function of the m^6^A reader Ythdf2, we generated conditional C57BL/6 *Ythdf2* targeted mice with LoxP sites flanking the 5′ UTR and exon 1 of the endogenous *Ythdf2* locus using CRISPR-Cas9 technology (Fig. 1a). The *Ythdf2*^*+/loxp*^ mice were crossed with mice ubiquitously expressing Cre-recombinase to generate the *Ythdf2*^+/−^ mice. Then to get *Ythdf2*^*−/−*^ mice, we intercrossed heterozygous *Ythdf2*^+/−^ mice. Interestingly, no viable *Ythdf2*^*−/−*^ newborn mice were identified in this particular knockout strain. The ratio of wild-type, hetero-, and homozygous knockout mice was not consistent with the expected 1:2:1 Mendelian ratio. Noteworthy, the number of postnatal *Ythdf2*^+/−^ mice indicated semi-lethality for these mice (Fig. 1b). Furthermore, 34% of *Ythdf2*^+/−^ surviving mice have malfunctioning eyes, with eyelids remaining closed (Additional file 1: Figure S1b). Many factors might contribute to this [29, 30], such as dysfunction of hypothalamic nerve control, but this was not studied further here.

To assess the stage of developmental failure, we collected embryos at E12.5 and E14.5 from heterozygote intercrosses and genotyped them by both PCR with primers flanking and inside the deleted genomic region (Fig. 1a, c) and western blotting with Ythdf2 antibody (Fig. 1d). PCR and western blot analysis confirmed that the expression of Ythdf2 is completely depleted in *Ythdf2*^*−/−*^ embryos. The Mendelian distribution of wild type, *Ythdf2*^+/−^, and *Ythdf2*^*−/−*^ was 1:2:1 when genotyped at embryonic stages E12.5–14.5 (Fig. 1b), suggesting the stage of embryonic lethality after E14.5. Therefore, we isolated embryos at E18.5 for further analysis. At this stage, 3 out of 41 embryos were genotyped as *Ythdf2*^*−/−*^ (data not shown). Despite the genotype ratio being normal at E12.5 and E14.5, the average number of embryos per litter was significantly less in *Ythdf2*^+/−^ intercrosses compared with wild-type intercrosses, especially at the late embryonic stage E18.5 (Fig. 1e). It was reported that removal of *Ythdf2* in zebrafish leads to 31.3% cell arrest and lethality at the one-cell stage by *Ythdf2*^+/−^ intercross matings [13], consistent with our finding of 30% less embryos at E12.5 and E14.5. According to our data, the major lethality of *Ythdf2*^*−/−*^ embryos occurred between E14.5 and E18.5. Therefore, disruption of the *Ythdf2* gene results in embryonic lethality during the late developmental stages of embryogenesis.

### *Ythdf2*^*−/−*^ mice display abnormal cortical development

**Fig. 2:**
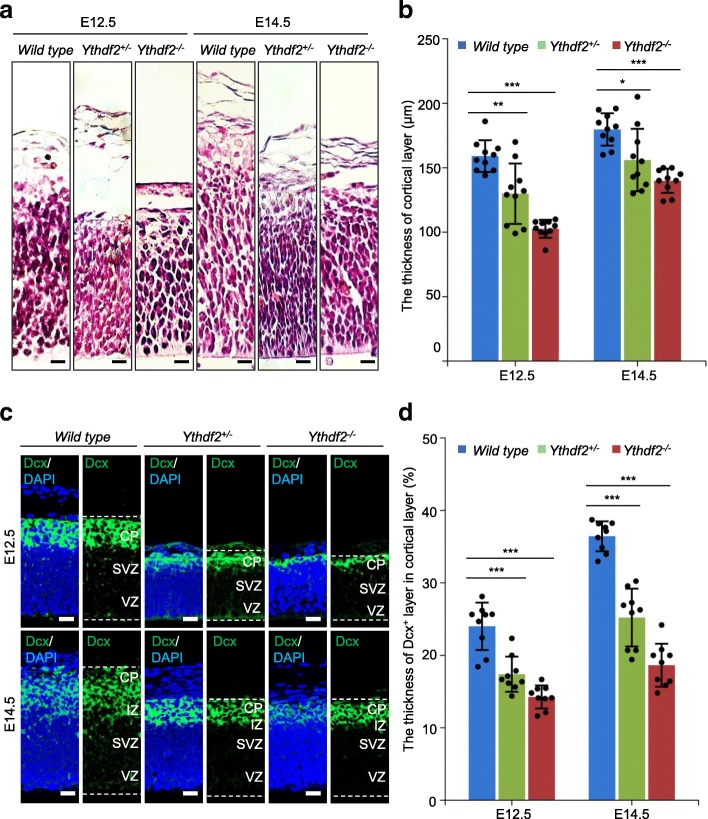
Ythdf2 is required for normal embryonic cortical development. **a** Sagittal brain sections of E12.5 and E14.5 were stained with H&E. An enlarged view of the forebrain cortex is shown. Scale bar indicates 20 μm. **b** Thickness of the cortical layer in *Ythdf2*^+/−^, *Ythdf2*^*−/−*^, and their wild-type littermates at E12.5 and E14.5. *Error bars* represent mean ± standard deviation, *n* = 3 embryos and 3 technical replicates. **c** Immunostaining of E12.5 and E14.5 brain sagittal sections for Dcx in wild-type, *Ythdf2*^+/−^, and *Ythdf2*^*−/−*^ littermates. Nuclei were counterstained with DAPI. *VZ* ventricular zone, *SVZ* subventricular zone, *IZ* intermediate zone, *CP* cortical plate. **d** Ratio of the thickness of Dcx-immunolabeled neuronal layers over cortical layers in *Ythdf2*^+/−^ and *Ythdf2*^*−/−*^ compared with wild type. *Error bars* represent mean ± standard deviation, *n* = 3 embryos and 3 technical replicates. *P < 0.05, **P < 0.01, ***P < 0.001, Student’s *t*-test

To determine how depletion of *Ythdf2* affects embryonic development, we dissected embryos at E12.5, E14.5, and E18.5. Although *Ythdf2*^*−/−*^ embryos at E12.5 and E14.5 were alive and appeared normal, sagittal sectionings of the whole embryos and H&E staining uncovered dramatically decreased overall cortical thickness of *Ythdf2*^*−/−*^ embryonic fore brains (Fig. 2a). Compared with their wild-type littermates, there was a general 56 μm decrease in the cortical layer at E12.5 and 40 μm decrease in the cortical layer at E14.5, yet the cortexes of both genotypes grew from E12.5 to E14.5 (Fig. 2b). The *Ythdf2*^+/−^ mice are semi-lethal. Thus, we also analyzed a cohort of *Ythdf2*^+/−^ mice and found a mean 29 μm decrease in the cortical layer at E12.5 and a mean 24 μm decrease in the cortical layer at E14.5 (Fig. 2a, b). We suspected that the delayed cortical development derived from a defect in the early stages of neurogenesis. In order to determine whether Ythdf2 expression is temporally associated with brain development, we analyzed the expression of *Ythdf2* in brain samples by quantitative RT-PCR at E12.5, E13.5, E17.5, and E18.5. *Ythdf2* was highly expressed during the early stage of neural development (Additional file 1: Figure S1a).

To further define the neuronal developmental failure associated with *Ythdf2* deficiencies, embryonic brain slices at different developmental stages were stained with the immature neuron marker doublecortin (Dcx). At E12.5, the neuronal layer of *Ythdf2*^*−/−*^ and *Ythdf2*^+/−^ embryos was significantly thinner than that of the wild type, here shown as the ratio of the thickness of Dcx-immunolabeled neuronal layers over cortical layers (Fig. 2c, d). Taken together, the in vivo evidence indicates a striking phenotype of retarded cortical development, resulting from decreased neurogenesis at the early stages of embryonic brain development.

### Basal progenitor cells are decreased in *Ythdf2*^*−/−*^ embryos

**Fig. 3:**
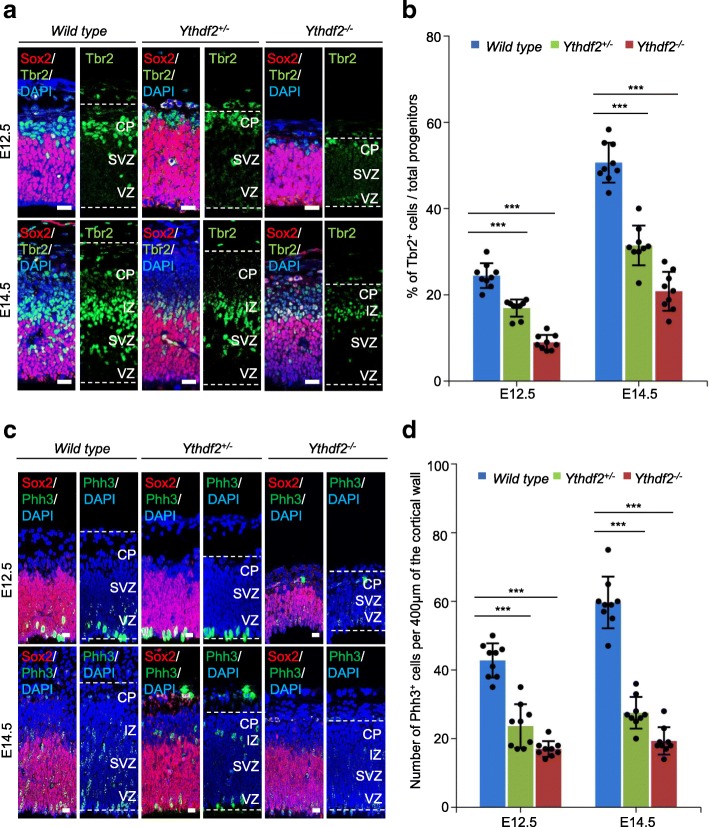
The number of basal progenitors and mitotic capability of apical progenitors depends on Ythdf2. **a** Immunostaining of E12.5 and E14.5 sagittal sections with Tbr2 (*green*) and Sox2 (*red*) antibodies in wild type, *Ythdf2*^+/−^, and *Ythdf2*^*−/−*^ embryos. *VZ* ventricular zone, *SVZ* subventricular zone, *IZ* intermediate zone, *CP* cortical plate. Nuclei were counterstained with DAPI. **b** Percentage of Tbr2^+^ cells over Tbr2^+^/Sox2^+^ at E12.5 and E14.5. *Error bars* represent mean ± standard deviation, *n* = 3 biological and 3 technical replicates. Scale bars, 20 μm. **c** Immunostaining of E12.5 and E14.5 sagittal sections with Phh3 (*green*) and Sox2 (*red*) antibodies in wild type, *Ythdf2*^+/−^, and *Ythdf2*^*−/−*^embryos. Nuclei were counterstained with DAPI. **d** Number of Phh3^+^ cells per 400 μm of the cortical wall at E12.5/E14.5 from **c**. *Error bars* represent mean ± standard deviation, *n* = 3 biological and 3 technical replicates. *P < 0.05, **P < 0.01, ***P < 0.001, Student’s *t*-test. Scale bars, 20 μm

Neural stem/progenitor cells (NSPCs) and immature neurons are the major cortical components at E12.5 and E14.5 in mice. NSPCs give rise to neurons. Given the profound effects of *Ythdf2* targeting on embryonic brain development, we examined the proliferation and differentiation capability of NSPCs during development. The T-box transcription factor Eomes (Tbr2) is specifically expressed in basal progenitor cells, predominantly in the SVZ, which primarily differentiate into superficial layer neurons. In E12.5 and E14.5 *Ythdf2*^*−/−*^ embryos and, to a lesser extent, *Ythdf2*^+/−^ embryos, there was a dramatic loss of basal progenitor cells, displayed by the obviously thinner Tbr2 layer, compared to wild type littermate embryos (Fig. 3a). The sex determining region Y-box2 (Sox2) is a marker for apical progenitor cells located in the VZ, which can produce deep layer neurons and basal progenitor cells [31]. The ratio of Tbr2-positive cells to total progenitors (Tbr2^+^/Sox2^+^) was decreased markedly at E12.5 and E14.5 in *Ythdf2*^*−/−*^ and *Ythdf2*^+/−^ embryos compared with the wild types (Fig. 3b), suggesting the decrease in neurons (Dcx^+^) associates with a reduction in the basal progenitor population in SVZ. However, there was no obvious difference in Sox2-positive apical progenitor cells in VZ layer (Fig. 3a).

### Mitotic capability of apical progenitor cells is impaired in *Ythdf2*^*−/−*^ embryos

The non-self-renewing basal progenitors only experience one or two mitotic cycles, and the majority of basal progenitors are established by asymmetric division of apical progenitor cells during early cortical development [32, 33]. We propose that the decrease in basal progenitors (Tbr2^+^) might be caused by the reduced mitotic capability of the Ythdf2-depleted apical progenitor cells. The E12.5 and E14.5 sagittal sections of wild type, *Ythdf2*^+/−^, and *Ythdf2*^*−/−*^ embryos were co-stained with the mitotic phase marker phospho-histone H3 (Phh3) and Sox2 to quantify the mitotic capability of the apical progenitor cells. The number of Phh3-positive cells decreased more than two-fold in *Ythdf2*^*−/−*^ cortex compared with wild type at E12.5 and E14.5. In *Ythdf2*^+/−^ cortex, the number of Phh3-positive cells was significantly reduced compared to the wild type cortex, yet was higher than in *Ythdf2*^*−/−*^ cortex (Fig. 3c, d). Additionally, apical progenitor cells could also maintain the population by several rounds of symmetric division in the VZ layer [34]. As there were no obvious changes in the number of apical progenitor cells (Sox2^+^) in the VZ layer, we concluded that Ythdf2-dependent defective neurogenesis was caused by the decreased generation of basal progenitors from apical progenitors.

### *Ythdf2*^*−/−*^ NSPCs exhibit decreased proliferation in vitro

**Fig. 4:**
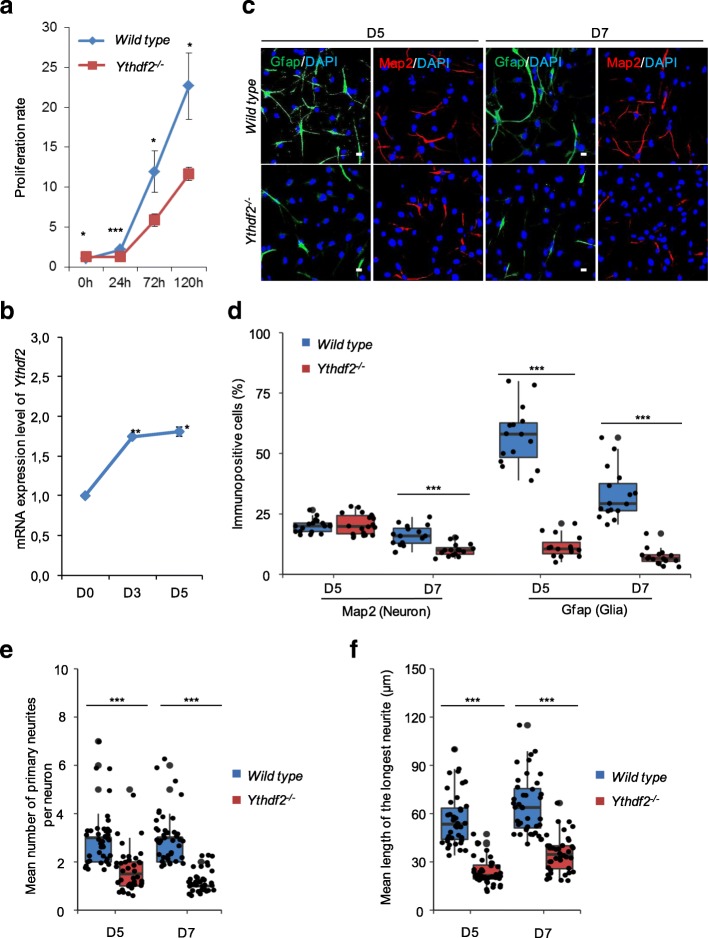
*Ythdf2*^*−/−*^ NSPCs exhibit decreased proliferation and defects in natural differentiation in vitro. **a** Number of viable NSPCs at 0, 24, 72, and 120 h monitored by signal intensity of Presto Blue reagent. Proliferation rate was calculated by normalizing to wild type at 0 h. **b** mRNA expression levels of *Ythdf2* during NSPC differentiation. Cells were collected at differentiation Day 0 (D0), 3, and 5. Isolated total RNAs were applied for RT-qPCR analysis. Actin was used as normalization control. **c** Immunostaining of Map2^+^ and Gfap^+^ cells differentiated from E14.5 neurospheres at D5 and D7. Nuclei were counterstained with DAPI. Scale bar indicates 20 μm. **d** Percentage of Map2 or Gfap positive cells. *Error bars* represent mean ± standard deviation, *n* = 3 biological repeats and 3 technical replicates. **e** Mean number of primary neurites per neuron (Map2^+^). *n* = 20 neurons for each biological repeat. **f** Mean length of the longest neurite of neurons (Map2^+^). *n* = 20 neurons for each biological repeat. *P < 0.05, **P < 0.01, ***P < 0.001, Student’s *t*-test

To further understand how Ythdf2 regulates neurogenesis, we cultured neurospheres consisting of NSPCs derived from E14.5 wild-type and *Ythdf2*^*−/−*^ embryonic fore brain. The *Ythdf2*^*−/−*^ neurospheres were smaller than the wild-type spheres (Additional file 1: Figure S2a, b). We first monitored the influence of Ythdf2 on NSPC proliferation. NSPCs dissociated from the primary neurospheres were seeded for proliferation testing and the cell growth was determined at 0, 24, 72, and 120 h. Compared with the wild type, *Ythdf2*^*−/−*^ NSPCs showed a slightly decreased proliferation rate after 24 h and a more pronounced reduction after 72 h culturing (Fig. 4a). This result is in agreement with the decreased mitotic capability of stem/progenitor cells observed in vivo.

### *Ythdf2*-deficient NSPCs show impaired neural differentiation

In differentiation assays, NSPCs dissociated from neurospheres produce both neurons and glial cells after 5 days culturing. We first assessed the mRNA expression profile of *Ythdf2* in wild-type neurospheres during differentiation by RT-qPCR. The expression of *Ythdf2* was up-regulated from Day 0 (D0) to D3 during differentiation and remained high till D5, suggesting the involvement of *Ythdf2* in regulating differentiation (Fig. 4b). Neuronal and glial cell lineages can be identified by staining with antibody against microtubule associated protein 2 (Map2) or glial fibrillary acidic protein (Gfap), respectively. We quantified the percentages of Gfap-positive cells for *Ythdf2*^*−/−*^ and wild-type at D5 and D7. Dramatic reduction of glial cells, with abnormal branches (Gfap^+^), was observed in differentiated *Ythdf2*^*−/−*^ neurospheres (Fig. 4c, d). However, we did not observe a significant different ratio of *Ythdf2*^*−/−*^ neurons (Map2^+^) at D5, while the ratio of *Ythdf2*^*−/−*^ neurons declined significantly more than the wild type at D7. These results were further substantiated by neuron progenitor antibody neuron-specific class III beta-tubulin (Tuj1) and glial progenitor antibody S100 calcium-binding protein B (S100-β) staining at D3 and D5 (Additional file 1: Figure S2c, d). At D3, the number of glial lineage progenitors had already declined in *Ythdf2*^*−/−*^ cells, while no difference was observed for neuronal lineage progenitor cells (Additional file 1: Figure S2c, d). The TUNEL assay showed significantly more dead *Ythdf2*^*−/−*^ cells, which might result from impaired differentiation (Additional file 1: Figure S2e, f).

### *Ythdf2*-deficient neurons display abnormal neurite outgrowth and increased sensitivity to arsenite

Whereas neuronal lineage differentiation (Map2^+^ or Tuj1^+^) was not affected at D5 in *Ythdf2*^*−/−*^ cells, the morphological analysis of Map2-positive cells showed that *Ythdf2*^*−/−*^ differentiated neurons had less and shorter primary neurites (axons and dendrites). The mean number of branching neurites per neuron in *Ythdf2*^*−/−*^ differentiated cells is less than in the wild type (Fig. 4e), and the mean length of the longest neurite in *Ythdf2*^*−/−*^ differentiated cells is shorter than in the wild type (Fig. 4f). The neurite outgrowth is pivotal in neuronal development and maturation, synaptic formation, neuronal function, and functional recovery in diseases [35]. The severe effect on neurite branching and extension of *Ythdf2*^*−/−*^ neurons might also contribute to the defective neurogenesis during neural development.

Besides, we found that differentiated neurons in vitro were more sensitive to arsenite treatment. Arsenite was demonstrated to induce oxidative stress by generating ROS and depleting antioxidants in cell lines and mammalian brain [36, 37]. It is reported that after arsenite treatment, Ythdf2 can co-localize with P body to regulate mRNA decay [7]. We treated differentiated neurons with 5 μM arsenite for 24 h in vitro, followed by recovery in fresh medium for 24 h (Additional file 1: Figure S3a). For wild-type neurons, the mean length of neurites was shortened and the number of neurites reduced after 24-h arsenite exposure (Additional file 1: Figure S3b, c). However, after 24-h culture in fresh medium, the remaining neurites recovered to the original length and the neurite number partially increased as the growth of new neurites needs longer time (Additional file 1: Figure S3b, c). In contrast, *Ythdf2*^*−/−*^ neurons showed increased sensitivity to arsenite exposure compared to wild-type neurons. After 24-h recovery, *Ythdf2*^*−/−*^ neurites could not outgrow to the original length and no new neurites projected.

### Negative regulation of neural development pathways enriched in *Ythdf2*^*−/−*^ neurospheres

To address the molecular mechanism of modulating NSPC proliferation and differentiation, we performed mRNA sequencing in the wild-type and *Ythdf2*^*−/−*^ neurospheres with three biological replicates. We identified 2144 up-regulated differentially expressed genes (DEGs) and 1756 down-regulated DEGs (Additional file 1: Figure S4a). With more stringent criteria (fold change > 1.5, *P* < 0.05 in three replicates), 151 significantly up-regulated and 316 significantly down-regulated genes were identified in *Ythdf2*^*−/−*^ neurospheres. Interestingly, the up-regulated genes were significantly associated with axon guidance, synapse assembly, neuron differentiation, and apoptosis. All these biological processes are subordinate to nerve development (Additional file 1: Figure S4b). The JAK-STAT signaling pathway is up-regulated in neurons and glial cells, which contributes to the neuroprotection and neurite outgrowth [38, 39]. The genes, highly enriched for Gene Ontology (GO) term “negative regulation of JAK-STAT cascade”, inhibit this cascade, such as *Flrt2*, *Flrt3*, *Ptprd*, and *Lrrtm1* and *4*. On the contrary, clustered terms, such as “positive regulation of cell differentiation”, “positive regulation of transcription”, “positive regulation of GTPase activity”, and “negative regulation of neuron apoptotic process”, were dominant in down-regulated genes.

### m^6^A-methylomes in wild-type and *Ythdf2*^*−/−*^ neurospheres

**Fig. 5:**
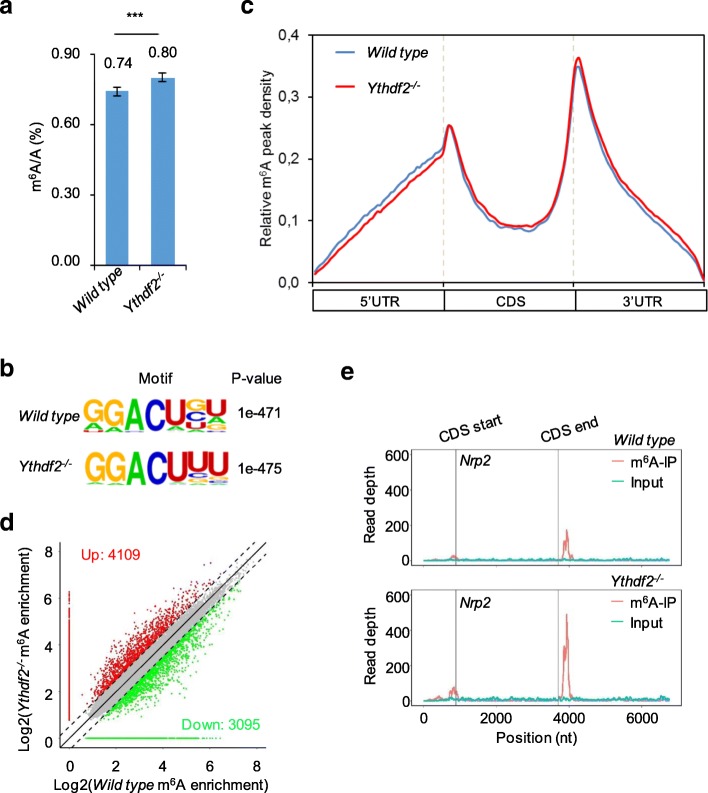
Overview of m^6^A methylomes in wild-type and *Ythdf2*^*−/−*^ neurospheres. **a** The m^6^A contents of mRNAs isolated from wild type and *Ythdf2*^*−/−*^ were quantified by LC-MS/MS. **b** Sequencing motif in m^6^A peaks verified in wild type and *Ythdf2*^*−/−*^ with HOMER database. **c** Distribution of m^6^A peaks along transcripts in wild type and *Ythdf2*^*−/−*^. **d** Scatter plot showing m^6^A peaks with increased (*red*) or decreased (*green*) levels. **e** Representative m^6^A distribution along Nrp2 transcript. Enrichment coverage of m^6^A and input are displayed as *red* and *blue*, respectively. *Grey lines* define coding sequence (*CDS*) borders

In order to gain more insight about the role of *Ythdf2*^*−/−*^ in m^6^A mRNA decay, we compared the m^6^A methylome of wild-type and *Ythdf2*^*−/−*^ neurospheres. Initially, we quantified the m^6^A/A ratio of the total mRNAs purified from the wild-type and *Ythdf2*^*−/−*^ neurospheres by LC-MS/MS. In *Ythdf2*^*−/−*^ neurospheres, the m^6^A abundance was increased by around 10% on average compared with the wild type (Fig. 5a). This is consistent with the m^6^A-dependent RNA decay function of Ythdf2 [7] and correlates very well with a study in zebrafish on the role of Ythdf2 in the maternal-to-zygotic transition [23]. We identified 16,626 common m^6^A sites from 8201 genes and 17,734 common m^6^A sites from 8585 genes in three biological replicates of wild-type and *Ythdf2*^*−/−*^ neurospheres, respectively (Additional file 1: Figure S5a). The highly over-represented m^6^A RRACH (R = G/A, H=U/A) motif identified using the HOMER algorithm in both wild-type (*P* = 1e-471) and *Ythdf2*^*−/−*^ (*P* = 1e-475) neurospheres proved the successful enrichment of m^6^A-modified mRNA (Fig. 5b and Additional file 1: Figure S5b). The m^6^A sites were significantly enriched at start codons, stop codons, and 3′ UTRs. The m^6^A profile is thus in very good agreement with those reported previously (Fig. 5c and Additional file 1: Figure S6a).

Based on the statistics from three biological replicates, 3095 m^6^A sites from 2464 genes and 4109 m^6^A sites from 2619 genes were identified to have lower or higher m^6^A levels in three biological replicates of *Ythdf2*^*−/−*^ neurospheres (Fig. 5d and Additional file 1: Figure S6b). m^6^A sites with significantly higher enrichment (fold change > 1.5) in all three *Ythdf2*^*−/−*^ replicates were analyzed further. Based on this stringent criterion, 78 m^6^A sites from 69 genes were markedly up-regulated. These genes were enriched for functional clusters like transcription regulation, phosphorylation, and neuron projection development (Additional file 1: Figure S7a). On the other hand, 102 m^6^A sites from 99 genes were down-regulated. These genes were enriched for functional clusters like transcription regulation, transport, rhythmic process, and apoptosis (Additional file 1: Figure S7b). Among these 168 genes, 115 genes had conserved m^6^A sites across samples, while 65 and 54 genes had newly occurring or absent m^6^A sites, respectively, in all three *Ythdf2*^*−/−*^ neurospheres (Additional file 1: Figure S7c).

### Ythdf2 is required for degradation of genes related to neuron differentiation

**Fig. 6:**
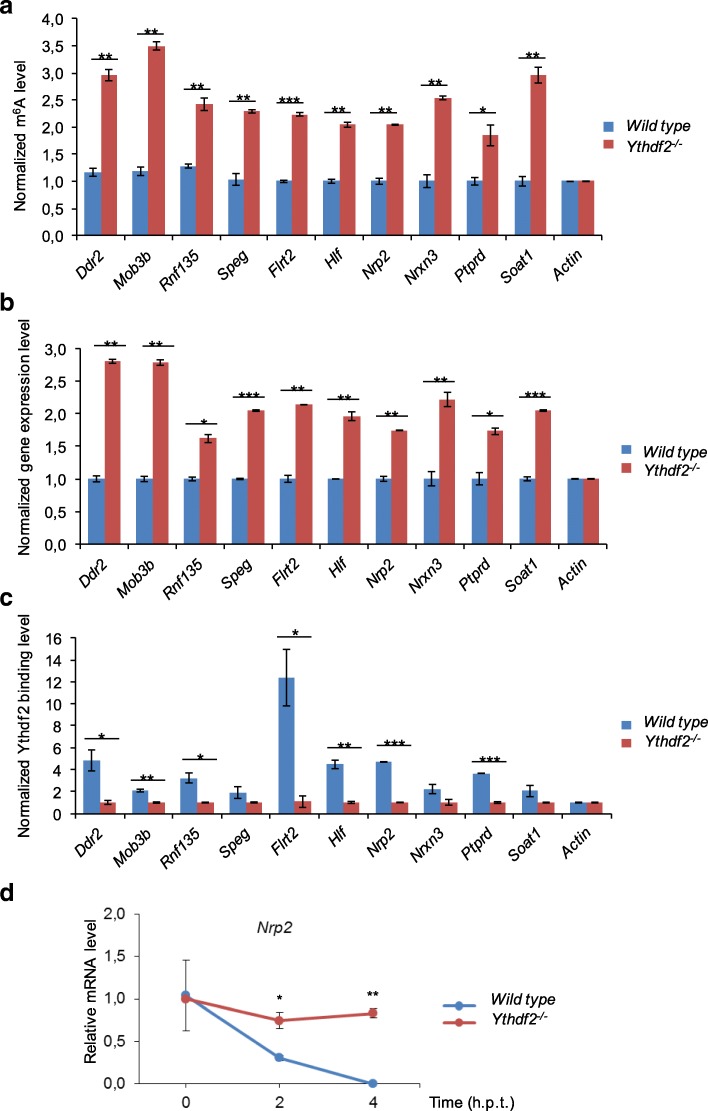
Ythdf2 is required for regulating mRNA decay of m^6^A-modified neuron-related gene targets. **a** m^6^A enrichment of target sites in gene candidates, verified by m^6^A IP combined with RT-qPCR. Non-m^6^A-modified gene *Actin* was used as negative control. **b** Gene expression of gene candidates, verified by RT-qPCR with input RNA. Non-changed gene *Actin* was used as negative control. **c** Ythdf2 binding levels of gene candidates, verified by Ythdf2 RIP combined with RT-qPCR. Non-m^6^A-modified gene *Actin* was used as negative control. **d** Representative mRNA profile of *Nrp2* at 0-, 2-, and 4-h time points after actinomycin D (5 μg/ml) treatment (*h.p.t.*) in wild type and *Ythdf2*^*−/−*^. *Error bars* represent mean ± standard deviation, *n* = 2 biological replicates. *P < 0.05, **P < 0.01, ***P < 0.001, Student’s *t*-test

It is well established that Ythdf2 specifically binds mRNAs containing m^6^A and promotes mRNA decay [1, 7]. In Ythdf2-depleted zebrafish embryos, Ythdf2-targeted mRNAs had extended lifetimes as seen by increased mRNA levels [13]. Hence, we focused on verifying candidate genes with increased mRNA transcripts and enrichment of m^6^A sites. Among these genes, *Nrp2* and *Nrxn3* were involved in nerve development and cell differentiation; *Flrt2* and *Ptprd* were enriched in negative regulation of JAK-STAT cascade, regulation of synapse assembly and axon guidance, and neuron differentiation; *Ddr2* was related to fibroblast proliferation; *Hlf* was involved in rhythmic process; and *Nrp2* and other genes showed enrichment of representative m^6^A peaks in *Ythdf2*^*−/−*^ neurospheres (Fig. 5e and Additional file 1: Figure S8). To further substantiate these findings, we performed m^6^A immunoprecipitation (IP) combined with RT-qPCR. Consistent with our initial findings, m^6^A IP showed that m^6^A levels increased significantly, while non-methylated actin was used as negative control (Fig. 6a). RT-qPCR showed that target genes were markedly enriched in *Ythdf2*^*−/−*^ neurospheres compared with the wild type (Fig. 6b). Next, we analyzed whether these m^6^A-enriched genes are real Ythdf2 targets by RNA IP (RIP) analysis. We confirmed that the Ythdf2 antibody was applicable to IP (Additional file 1: Figure S9). Compared with *Ythdf2*^*−/−*^, Nrp2 mRNA and other candidates were enriched by Ythdf2 protein in the wild type, which was verified by qPCR (Fig. 6c). To examine whether increased gene expression was due to loss of Ythdf2-mediated RNA decay, we measured the mRNA life time of these candidate genes by inhibition of transcription with actinomycin D in wild-type and *Ythdf2*^*−/−*^ neurospheres. After actinomycin D treatment, mRNA levels of *Nrp2* and the other candidate genes in wild type declined more rapidly than in *Ythdf2*^*−/−*^ neurospheres (Fig. 6d and Additional file 1: Figure S10). Thus, the increased levels of m^6^A-modified mRNA transcripts in the absence of Ythdf2 were caused by delayed mRNA clearance, which might contribute to the defects in neurogenesis.

## Conclusions

Ythdf2 is essential for oocyte maturation and early zygotic development in zebrafish and mouse [13, 25]. Ythdf2 was recently reported to be required for oocyte competence through the post-transcriptional regulation of the maternal transcriptome and homozygous *Ythdf2*^*−/−*^ mice were reported to be partially permissive at weaning, with approximately 80% loss of homozygous *Ythdf2*^*−/−*^ mice in inbred C57BL/6 mice [25]. The targeting of the *Ythdf2* locus described here caused a complete loss of homozygous *Ythdf2*^*−/−*^ mice in our inbred C57BL/6 background and the majority of *Ythdf2*^*−/−*^ embryos died at late embryonic stages (Fig. 1). Of note, intercrossing *Ythdf2*^+/−^ mice results in constantly smaller litter size than from wild type matings (Fig. 1e), indicating an essential role of Ythdf2 in early embryo development. Here we reveal a crucial role of m^6^A in mRNA and its binding protein Ythdf2 in neural development at embryonic developmental stages. The mammalian nervous system arises from the ectoderm, with both neurons and glial cells (astrocytes and oligodendrocytes) generated from NSCs in a precisely regulated spatiotemporal order [34]. We propose that erroneous recognition and degradation of m^6^A-containing mRNA at this stage leads to the dysregulation of neural development.

The m^6^A level in mRNAs is higher in brain than in other studied mouse organs, indicating a crucial role during normal brain development [11]. Recent studies found m^6^A-modifying enzymes Mettl3, Alkbh5, and Fto to be involved in regulating progression of glioblastoma, indicating that m^6^A epitranscriptomic regulation plays roles in the nervous system. Very recently, Yoon et al. [27] used a methyltransferase Mettl3-Mettl14 complex knockout to demonstrate that m^6^A depletion extends cortical neurogenesis by protracting cell cycle progression of NPCs. In this study, we demonstrate the severe impact of *Ythdf2* deletion on corticogenesis, neurogenesis, and gliogenesis. During early neural development, the decreased thickness of cortex is attributed to the dramatically thinner CP and SVZ layers, composed of neurons (Dcx^+^) and basal progenitor cells (Tbr2^+^), respectively (Figs. 2c and 3a). Multiple factors are supposed to contribute to this. First, consistent with the documented function of m^6^A in proliferation of NPCs [27], our in vivo and in vitro evidence reveals that the proliferation capability of the NSPCs is severely compromised in *Ythdf2*^*−/−*^ embryonic cortex NSPCs. Further, apical progenitors symmetrically divide into more apical progenitors to expand the stem cell/progenitor VZ pool [34]. No significant change in the thickness of the VZ layer in *Ythdf2*^*−/−*^ embryos suggests that the symmetric division of apical progenitors is not disturbed. However, apical mitosis is significantly decreased in *Ythdf2*^*−/−*^ embryos. Apical progenitor cells can give rise to basal progenitor cells and neurons by asymmetric division [40, 41]. The switch between symmetric and asymmetric cell division of *Ythdf2*^*−/−*^ neural progenitor cells, which determines self-renewal or differentiation, may be disturbed. It is worth mentioning that it is not confirmed that the neural defects observed contribute to embryonic lethality. So it will be interesting to address this relationship by generating neural-specific Ythdf2 knockout mice. Second, the NSPCs derived from *Ythdf2*^*−/−*^ embryo brains generated similar numbers of neurons as wild type in vitro (Fig. 4c and Additional file 1: Figure S2c). However, morphological analysis demonstrated abnormal neurite outgrowth of *Ythdf2*^*−/−*^ neurons that are more vulnerable to stress and fail to recover from neurite degeneration (Fig. 4e, f and Additional file 1: Figure S3). Proper neurite outgrowth and branching is pivotal for establishing neuronal circuits which facilitate nervous system function [42]. Interestingly, RNA-seq analysis shows that differentially expressed genes (DEGs) relate to functions such as axon regulation, synapse assembly, and neuron differentiation (Additional file 1: Figure S4). Among them, genes such as *Ddr2*, *Rnf135*, *Flrt2*, *Hlf*, *Nrp2*, *Nrxn3*, and *Ptprd* have both up-regulated mRNA and m^6^A levels (Fig. 6a, b). Ythdf2-mediated mRNA decay affects the translation efficiency and lifetime of m^6^A-modified mRNA targets [7]. By recruiting the Ccr4-not deadenylase complex, Ythdf2 initiates the degradation of its mRNA targets at specialized decay sites [43]. The RIP combined RT-qPCR and mRNA life-time assays display that mRNA levels of these genes are stabilized due to the complete absence of Ythdf2 in *Ythdf2*^*−/−*^ NSPCs (Fig. 6c, d and Additional file 1: Figure S10). Delayed mRNA degradation causes the retention of m^6^A-modified transcripts in *Ythdf2*^*−/−*^ neurospheres, leading to increased m^6^A enrichment.

Last but not least, while homozygous *Ythdf2*^*−/−*^ is embryonic lethal, heterozygous *Ythdf2*^+/−^ is unexpectedly only partially lethal, with 30% of the surviving *Ythdf2*^+/−^ mice having eye defects (Additional file 1: Figure S1b), which may reflect haploid insufficiency of *Ythdf2* and a malfunctioning nervous system. Furthermore, m^6^A is highly enriched in mouse brain, and the level is dramatically increased with postnatal aging [11]. Taken together, we propose that m^6^A and Ythdf2 have a pivotal function in brain not only during embryonic neural development but also in postnatal life. Thus, functions of m^6^A and Ythdf2 on postnatal nervous system development merits further investigations.

During revision of this manuscript, two studies relating to the role of m^6^A in the adult mammalian nervous system were reported. One study found that either the m^6^A methyltransferase Mettl14 or the m^6^A-binding protein Ythdf1 regulate functional axon regeneration in the peripheral nervous system in vivo by modulating injury-induced protein translation [44]. In another study it was discovered that m^6^A in mRNA regulates histone modification in part by destabilizing transcripts that encode histone-modifying enzymes, which might be a previously unknown mechanism of gene regulation in mammalian cells [45].

In summary, our study demonstrates a pivotal function of Ythdf2-mediated m^6^A epitranscriptomic regulation in cortical neurogenesis during embryonic neural development, via regulating RNA degradation of m^6^A-tagged genes associated with neural development and differentiation.

## Methods

### Generation of conditionally *Ythdf2* gene-targeted mice

The Ythdf2 conditional knockout mouse model (mYthdf2-CKO) was generated as described in Additional file 1: Figure S1a by Applied Systemcell Inc. (CA, USA) using CRISPR-Cas9 technology. A cocktail of active guide RNA molecules (gRNAs), two single-stranded oligo donor nucleotides (ssODNs) and qualified Cas-9 mRNA was microinjected into the cytoplasm of C57BL/6 embryos. Two LoxP sites were inserted, flanking the upstream of 5′ UTR and intron 1 regions, resulting in loss and changes in size of PCR products. *Ythdf2*^*fl/fl*^ mice were genotyped and further sequenced for the LoxP cassettes at the designated locations. Potential *Ythdf2-CKO* mice were generated by crossing *Ythdf2*^*fl/fl*^ mice with Cre_Del_GT_07 mice from the Norwegian Transgenic Center (NTS, Oslo, Norway).

For Ythdf2 genotyping, ear-clip samples were lysed in alkaline lysis reagent (25 mM NaOH, 0.2 mM EDTA, pH 12) at 95 °C for 30 min, followed by adding neutralization reagent (40 mM Tris-HCl, pH 5). PCR conditions for wild type and knockouts: 95 °C, 2 min, 1 cycle; 95 °C, 30s; 60 °C, 30s; 72 °C, 1 min; 35 cycles. The PCR products were described in Additional file 1: Figure S1b. Primers for genotyping were as follows: wild-type allele (WT), 5′-TACGGGTGAGGTGTCTTTTTCTT-3′, 5′-GAAAGAGAGGAAACGAGGAAG-3′; targeted allele (KO), 5′-GGCTCTCCCTTCCCGAGAT-3′, 5′-GCTTTTGTCCCTGACACTCG-3′.

### Antibodies

The following antibodies were used at the appropriate dilutions: mouse anti-Map2 (M4403, Sigma), rabbit anti-Gfap (Z0334, DAKO), mouse anti-Tuj1 (MAB1195, R&D Systems), rabbit anti-s100-β (ab52642, Abcam), rabbit anti-Dcx (ab18723, Abcam), rabbit anti-Tbr2 (ab23345, Abcam), rabbit anti-phospho-Histone H3 (PHH3; Ser10; 06–570, Millipore), mouse anti-Sox2 (ab79351, Abcam), mouse anti-Nestin (MAB353, Millipore), rabbit anti-Ythdf2 (RN123PW, MBL), mouse anti-anti-β-actin (A1978, Sigma).

### RT-qPCR analysis

The total RNA was isolated using TRIzol LS Reagent (Life Technologies, 10,296–010). Normally, 1 μg total RNA was used for reverse transcription using High-Capacity cDNA Reverse Transcription Kit (ThermoFisher, 4,368,814). The quantitative PCR reactions were carried out with Power SYBR Green PCR Master Mix (Life Technologies, 4,368,708) on a StepOnePlus™ Real-Time PCR System instrument (Applied Biosystems). Primers used in this study were as follows.

**Table:** 

**Primers**	
*Ddr2*	Forward, TTGGCCACCCAAACAATCCA Reverse, AGACCCCTCTGGTCACCAAC
*Mob3b*	Forward, GAAAGCGATCCTGACTTCCAG Reverse, GCTAGCAGCACTTAGAGGGT
*Rnf135*	Forward, ACTGGGAAGTGGACACTAGG Reverse, CCAGGAGTCCATAGTCCTTCC
*Speg*	Forward, CTAGTGGTGCGGGCAAATCT Reverse, CCTGGTTAGCGGGAATTGGT
*Flrt2*	Forward, GACTGCCACATCCCCAACAA Reverse, CACCTTTCTAACGCTGGACCT
*Hlf*	Forward, CTGAAGGAGAACCAGATCGCA Reverse, TTCTTGCATTTGCCCAGCTC
*Nrp2*	Forward, CCCTTTGGAAACTGAATGCCA Reverse, GATCCCCTTCACAGCTGCAT
*Nrxn3*	Forward, ACGTATGGGCTCCATTTCCT Reverse, TTCTTGAGGCTTCCCGTGAG
*Ptprd*	Forward, TGAGCCATACAGGGCACTTG Reverse, GCCTCCTAAGTCAGGATTCTTGT
*Soat1*	Forward, GTGCAAGGGTGAGCCTATGT Reverse, GTGTGAGCAACTTGTACGGC
*Actin*	Forward, TTCTTTGCAGCTCCTTCGTT Reverse, ATGGAGGGGAATACAGCCC

### Western blotting

Total protein lysate was extracted with RIPA buffer (20 mM Tris-HCl, pH 7.4, 20% glycerol, 0.5% NP40, 1 mM MgCl_2_, 150 mM NaCl, 1 mM EDTA, 1 mM EGTA). Protein concentrations were measured using the Bradford Assay, and 50–100 μg protein extracts were subjected to SDS-PAGE. Then proteins were transferred to a nitrocellulose membrane, blocked with 5% non-fat milk and incubated with first antibodies for 1 h at room temperature. After incubation with secondary antibody against mouse (1:10,000) or rabbit (1: 10,000) for 1 h at room temperature, the membrane was visualized with an ECL Western Blotting Detection Kit (32,106, Thermo).

### Immunohistochemistry and immunofluorescence

For immunohistochemistry, embryonic brain tissues were dissected in cold PBS and fixed in 4% PFA at 4 °C for 48 h. Slides (4 μm thick) were sectioned by microtome (HM355s, Thermo Scientific) and deparaffinized and cleared in Clear-Rite™ 3 (6901TS, Thermo) followed by rehydration in an EtOH gradient. After antigen retrieval in citrate buffer (pH 6.4), the slides were blocked with blocking buffer (5% goat gut, 5% BSA, 0.1% tween-20, 0.5% Triton X-100) for 1 h, and incubated with primary antibodies overnight at 4 °C. Secondary antibodies were applied at room temperature for 1 h. For immunofluorescence, cultured cells were fixed with 4% paraformaldehyde (PFA), permeabilized with 0.1% Triton X-100, and stained with primary antibodies and secondary antibodies. Nuclei were visualized with mounting medium with DAPI (BioNordika, H-1200). Images were taken with a Leica SP8 confocal microscope equipped with a ×40 oil immersion lens.

### H&E staining

Tissue slides were stained in haematoxylin (Richard-Allen Scientific, 12,687,756) and eosin (Nerliens Meszansky, 161,170) after dehydration and rehydration, followed by differentiation in acetic acid in 100% ethanol at 1:50,000 dilution for 5 s. Then, the sections were dehydrated in ascending series of ethanol, treated with xylene, and coverslipped using Cytoseal XYL xylene-based mounting medium (8312–4, Thermo). Images were taken with a Zeiss AxioPlan 2 microscope system.

### TUNEL assay

Cells were grown on coverslips, fixed on ice with 4% PFA for 10 min, and permeabilized with 0.2% Triton X-100 in PBS-Tween for 30 min on ice. After incubating in 3% H_2_O_2_ in PBS for 10 min, slides were rinsed twice with PBST. Slides were incubated with 50 μl TUNEL reaction mixture for 60 min at 37 °C. Nuclei were visualized with mounting medium with DAPI (BioNordika, H-1200). Images were taken with a Leica SP8 confocal microscope equipped with a × 40 oil immersion lens.

### Neurosphere proliferation and differentiation

Neurospheres derived from E14.5 embryonic fore brains were cultured with DMEM/F12 (GIBCO) supplemented with 20 ng/ml EGF (R&D Systems, 236-EG-200), 10 ng/ml bFGF (R&D Systems, 234-FSE 025), N2 supplement (Life, 17,502–048), and B27 supplement without vitamin A (Thermo, 12,587,010). Under the proliferating condition, cells were grow as free-floating neurospheres. For secondary neurosphere formation, cells in primary neurospheres were trypsinized with TrypLE™ Express Enzyme (Gibco, 12,604,021) combined with DNaseI (Thermo, 18,047,019), dissociated mechanically by pipetting onto a six-well plate at 5 × 10^5^ cells per well. For the neurosphere differentiation assay, a set of neurospheres were trypsinized to obtain a suspension of dissociated cells. These cells were then plated in tissue culture plates pre-coated with poly-L-lysine (Sigma, P6516). Cells were cultured in differentiation medium (minus EGF and bFGF) and collected at different time points.

### Proliferation assay

Dissociated single NSPCs were seeded at a density of 1.0 × 10^4^ per well in 96-well plates. The proliferation rates were measured at 24, 72, and 120 h with PrestoBlue Cell Viability reagent (A13262, ThermoFisher Scientific) as instructed.

### Ythdf2 RIP

Ythdf2 RIP was carried out with a modified procedure [46]. Briefly, 1 × 10^7^ collected NSPCs were lysed in NETN buffer (20 mM Tris-Cl, pH 8.0; 100 mM NaCl, 1 mM EDTA, 0.5% NP-40, freshly added protease inhibitor cocktail and RNasin) for 20 min on ice. After centrifugation, the supernatant containing the RNA–protein complex was incubated with 5 μg Ythdf2 antibody (RN123PW, MBL) for 2 h at 4 °C. Then 30 μl Dynabeads G beads were added and rotated for 2 h, at 4 °C. Beads were collected with a magnetic stand and washed with NETN buffer four times. The RNA–protein complex was eluted by incubating with NETN buffer with 0.1% SDS and 30 μg proteinase K at 50 °C for 30 min. RNAs were further purified with RNA Clean and Concentrator-5 (Zymo).

### LC-MS/MS

Purified mRNA was digested by nuclease P1 (2 U, Wako) in 25 μl of buffer containing 10 mM of NH_4_OAc (pH 5.3) at 42 °C for 2 h, followed by the addition of NH_4_HCO_3_ (1 M, 3 μl, freshly made) and alkaline phosphatase (0.5 U). After an additional incubation at 37 °C for 2 h, the sample was diluted to 50 μl and filtered (0.22 μm pore size, 4 mm diameter, Millipore), and 5 μl of the solution was subjected to LC-MS/MS. Nucleosides were separated by reverse-phase ultra-performance liquid chromatography on a C18 column with on-line mass spectrometry detection using an Agilent 6410 QQQ triple-quadrupole LC mass spectrometer in positive electrospray ionization mode. The nucleosides were quantified using the nucleoside to base ion mass transitions of 282 to 150 (m^6^A) and 268 to 136 (A). Quantification was performed in comparison with the standard curve obtained from pure nucleoside standards running on the same batch of samples. The ratio of m^6^A to A was calculated based on the calibrated concentrations.

### mRNA isolation and m^6^A-RIP

NSPCs (1 × 10^7^) dissociated from neurospheres were collected for total RNA isolation with Direct-zol RNA miniprep plus with TRI Reagent (Zymo research, R2073) and DNase I digestion following the manufacturer’s instructions. We applied 1 mg total RNA for further mRNA purification with a Dynabeads mRNA DIRECT™ purification kit (Thermo, 61,011) for two rounds. The mRNA quality was checked using a 2100 Bioanalyzer instrument with an Agilent RNA 6000 Nano kit (5067–1511).

RNA fragmentation (1 μg) was performed by sonication at 10 ng/μl in 100 μl RNase-free water with Bioruptor Pico (Diagenode) with 30 cycles of 30 s on followed by 30 s off; 5% of the fragmented RNA was saved as input. m^6^A IP was performed with an EpiMark® *N*^*6*^-Methyladenosine Enrichment Kit (NEB, E1610S) following the kit manual adapted for the KingFisher™ Duo Prime Purification System. In detail, 1 μl N6-methyladenosine antibody from the kit and 25 μl Protein G beads (NEB #S1430) were used for each affinity pull down. After incubating with RNA, the beads were washed with 200 μl low salt reaction buffer twice, and then 200 μl high salt reaction buffer twice. RNA that was pulled down (IP) was eluted with 50 μl RLT buffer twice, and recovered by RNA Clean and Concentrator-5 (Zymo). Both input and IP were subjected to RNA library preparation with Truseq Stranded mRNA Library Prep Kit with the RFP incubation step shorten from 8 min to 20 s. Sequencing was carried out on Illumina HiSeq 4000 according to the manufacturer’s instructions.

### Sequencing data analysis

The sequencing data were mapped to mouse genome version mm10 downloaded from UCSC. Data analysis was carried out as previously described. Briefly, reads were aligned to mm10 using TopHat v2.0.142. For input analysis (RNA-seq), RPKM were calculated by Cuffnorm3. For m^6^A peak calling, the longest isoform was used if multiple isoforms were detected. Aligned reads were extended to 100 nucleotides (average fragment size) and converted from genome-based coordinates to isoform-based coordinates to eliminate interference from introns in peak calling. The longest isoform of each mouse gene was scanned using a 100-nucleotide sliding window with 10-nucleotide steps. To reduce bias from potentially inaccurate gene structure annotation and the arbitrary use of the longest isoform, windows with read counts less than 1/20 of the top window in both m^6^A IP and input samples were excluded. For each gene, the read count in each window was normalized by the median count of all windows of that gene. The window was called positive if FDR < 1% and log2(enrichment score) ≥ 1. Overlapping positive windows were merged. The following four numbers were calculated to obtain the enrichment score of each peak (or window): read count of the IP sample in the current peak/window (a); median read count of the IP sample in all 100-nucleotide windows on the current mRNA (b); read count of the input sample in the current peak/window (c); and median read count of the input sample in all 100-nucleotident windows on the current mRNA (d). The enrichment score of each window was calculated as (a × d)/(b × c). Common peaks shared in the triplicates of a sample were kept with the peak annotation from replicate 1.

For motif analysis, consensus motif was determined by using HOMER4. For GO analysis, differentially expressed genes or m^6^A-modified genes were uploaded to DAVID (http://david.abcc.ncifcrf.gov/). The GO terms were ranked and presented according to −log2(*P* value).

### mRNA life-time assay

Wild-type and *Ythdf2*^*−/−*^ neurospheres were trypsinized with TrypLE™ Express Enzyme (Thermo, 12,605,010). The dissociated cells were seeded into plates coated with PDL (Millipore, A-003-E) and laminin (R&D Systems, 3446–005-01). After 12-h culturing, cells were treated with 5 μg/ml actinomycin D (Sigma, A9415) for 2 and 4 h, while cells without treatment were used as 0 h. Cells were collected at designated time points and total RNA was extracted for reverse transcription and qPCR.

### Statistical analysis

All statistical analyses were performed with GraphPad Prism 5. Student’s *t*-test was adapted and data are shown as mean ± standard deviation. *P* value is used for significance.

## Additional file

Additional file 1: **Figures S1**–**S10.** This document contains additional supporting evidence for this study presented in the form of supplemental figures. (PDF 1160 kb)
