# Supplementary material for: Ythdf2-mediated m6A mRNA clearance modulates neural development in mice
Source: Genome Biol. 2018 May 31;19:69. doi: 10.1186/s13059-018-1436-y (PMC5984442; doi:10.1186/s13059-018-1436-y)
Supplement: Supplementary file 1 — Figures S1–S10. This document contains additional supporting evidence for this study presented in the form of supplemental figures. (PDF 1160 kb) [file 13059_2018_1436_MOESM1_ESM.pdf]

Additional file 1

Ythdf2-mediated m^6A mRNA clearance modulates neural development in mice

Miaomiao Li[1,2*], Xu Zhao[1,2*#], Wei Wang[3], Hailing Shi[4,5], Qingfei Pan[6], Zhike Lu[4,5], Sonia Peña Perez[1], Rajikala Suganthan[1], Chuan He[4,5,7], Magnar Bjørås[1,3#], Arne Klungland[1,2#]

[1] Department of Microbiology, Oslo University Hospital, Rikshospitalet, NO-0027 Oslo, Norway. [2] Department of Molecular Medicine, Institute of Basic Medical Sciences, University of Oslo, NO-0317 Oslo, Norway. [3] Department of Clinical and Molecular Medicine, Norwegian University of Science and Technology (NTNU), Trondheim, Norway. [4] Department of Chemistry, Department of Biochemistry and Molecular Biology, and Institute for Biophysical Dynamics, The University of Chicago, 929 East 57th Street, Chicago, Illinois 60637, USA. [5] Howard Hughes Medical Institute, The University of Chicago, 929 East 57th Street, Chicago, Illinois 60637, USA. [6] Department of Computational Biology, St. Jude Children's Hospital, Memphis, Tennessee 38105, USA. [7] Department of Biochemistry and Molecular Biology, The University of Chicago, 929 East 57th Chicago, Illinois 60637, USA.

**-These authors contributed equally*

#- Correspondence: Xu Zhao (Xu.xuzha@rr-research.no), Magnar Bjørås (magnar.bjoras@ntnu.no) and Arne Klungland (arne.klungland@medisin.uio.no)

Legends for Supplementary Figures S1-S10

Figure S1. *Ythdf2*$^{-/-}$ mouse displays malfunctioned eyes. **a** Temporal quantification of Ythdf2 mRNA levels by quantitative RT-PCR using beta actin mRNA as internal control. Error bars, mean ± s.d., n = 3 technical replicates, *P < 0.05, **P < 0.01, ***P < 0.001, Student's t-test. Scale bars, 20μm. **b** Frontal view and side view of eyes from *wild type* and *Ythdf2*$^{+/-}$ mice.

Figure S2. *Ythdf2*$^{-/-}$ NSPCs have defects in growth and differentiation. **a** The morphology of neurospheres which are cultured 3 days after passage. Scale bar indicates of 125 μm. **b** Diameters of the neurospheres. n > 500 colonies from 3 biological repeats. **c** Immunostaining of Tuj1^{+} and S100-β^{+} cells differentiated from E14.5 neurospheres at D3 and 5. Nuclei were counterstained with DAPI. Scale bar indicates of 125 μm. **d** Percentages of differentiated Tuj1 and S100-β positive cells from (C). n = 3 biological repeats. **e** Representative staining of apoptotic cells detected by TUNEL assay. Nuclei were counterstained with DAPI. Scale bar indicates of 80 μm. **f** Percentage of TUNEL positive cells from (E). n = 3 biological repeats. Error bars, mean ± s.d. *P < 0.05, **P < 0.01, ***P < 0.001, Student's t-test.

Figure S3. *Ythdf2*$^{-/-}$ neurons have abnormal neurite outgrowth and are sensitive to arsenite treatment. **a** Immunostaining of neurons (Map2^{+}) with or without arsenite treatment. Differentiated neurons are treated with 5 μM arsenite for 24h, followed by 24h recovery in fresh medium. Nuclei were counterstained with DAPI. Scale bar indicates of 80 μm. **b** Mean length of the longest neurite of neurons. n = 2 biological repeats and 2 technical repeats, 20 cells for each repeat. **c** Percentage of neurons with

different numbers of neurites. Error bars, mean ± s.d., n = 2 biological repeats and 2 technical repeats, 20 cells for each repeat. *P < 0.05, **P < 0.01, ***P < 0.001, Student's t-test.

Figure S4. DEG and GO analyses. **a** Scatter plot showing genes with increased or decreased expression levels. **b** Heat map showing expressions of significantly DEGs. The right panel showing GO terms enriched for up-regulated and down-regulated genes, respectively.

Figure S5. m^6A peaks in three biological repeats and motif analysis. **a** m^6A peaks identified in three biological repeats of *wild type* and *Ythdf2*$^{-/-}$. **b** Top three representative sequencing motifs in m^6A peaks verified in *wild type* and *Ythdf2*$^{-/-}$ with HOMER database.

Figure S6. Distributions of m^6A peaks. **a** Pie charts showing distributions of m^6A peaks in different regions of mRNA transcripts in *wild type* and *Ythdf2*$^{-/-}$. **b** Venn diagram depicts overlaps of m^6A peaks in three biological repeats.

Figure S7. GO analysis of significantly differentiated m^6A peaks. **a** Top 10 biological pathway GO terms enriched for genes with significantly up-regulated m^6A peaks. **b** Top 10 biological pathway GO terms enriched for genes with significantly down-regulated m^6A peaks. **c** The m^6A heat map showing distribution of m^6A sites in 5'UTR, start codon, CDS, stop codon and 3'UTR in *wild type* and *Ythdf2*$^{-/-}$. Blue lines represent m^6A sites. Each horizontal line represents one gene. The DEG heat map showing expressions of consistent genes from m^6A heat map in *wild type* and *Ythdf2*$^{-/-}$.

Figure S8. Profiles of m^6A peaks in Ythdf2 candidate targets. Representative m^6A peaks along candidate transcripts. Enrichment coverage of m^6A and Input were displayed as red and blue, respectively. Grey lines define CDS borders.

Figure S9. Validation of Ythdf2 antibody for immunoprecipitation. *Wild type* and *Ythdf2*$^{-/-}$ NSPCs were collected for protein immunoprecipitation (IP) with Ythdf2 antibody. Rabbit IgG was used as negative IP control. The same amount of pulled-down proteins were applied for western blot verification with Ythdf2 antibody. Actin was used as loading control.

Figure S10. mRNA levels of candidate genes after transcription is inhibited. mRNA profiles of candidate genes at 0, 2 and 4hr time points after actinomycin D (5 μg/ml) treatment (h.p.t.) in *wild type* and *Ythdf2*$^{-/-}$. Error bars, mean ± s.d., n = 2 biological repeats, $*P < 0.05$, $**P < 0.01$, $***P < 0.001$, Student's t-test.

Fig S1.

a

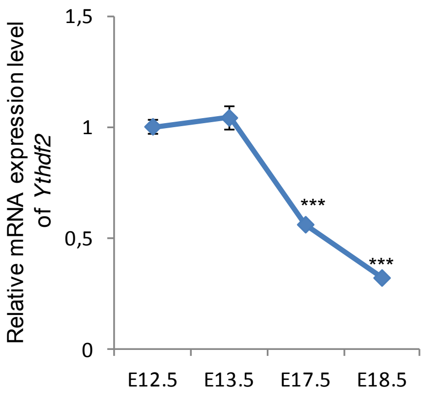

Relative mRNA expression level of Ythdf2
1,5
1
0,5
0
***
***
E12.5
E13.5
E17.5
E18.5

b

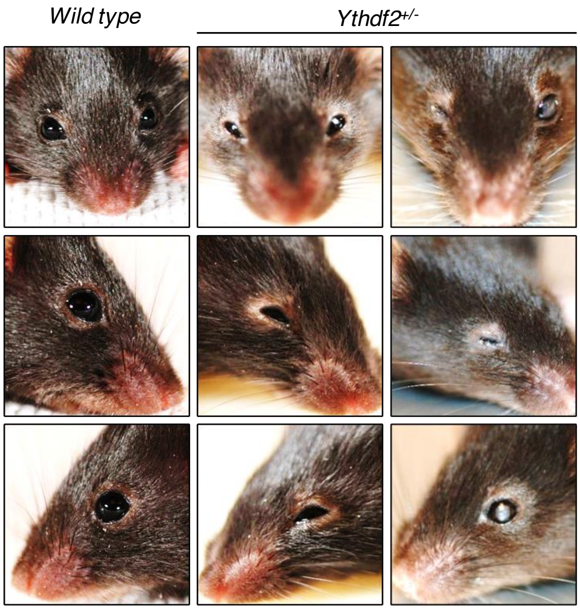

Wild type
Ythdf2+/-

Fig S2.

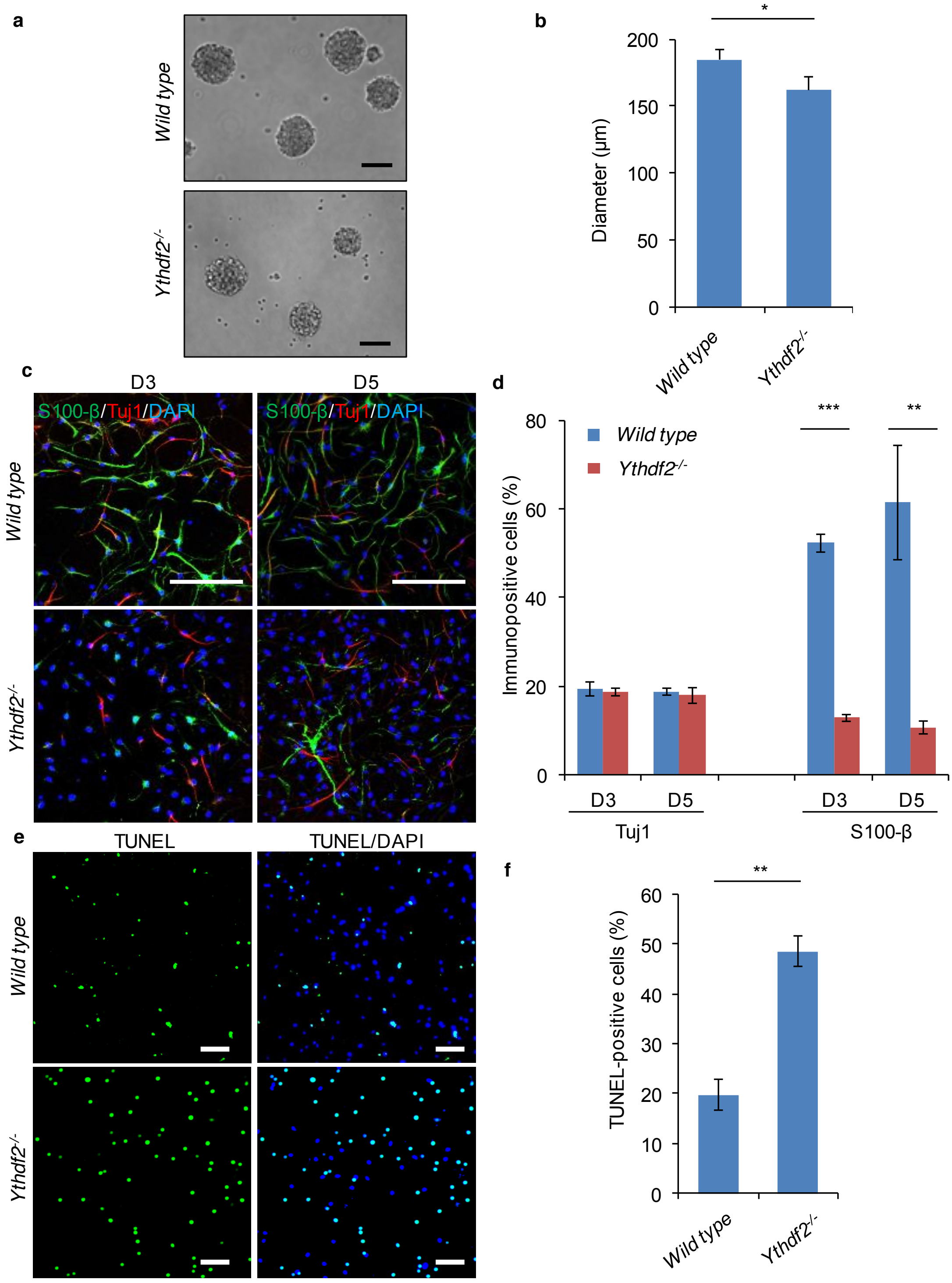

a
Wild type
Ythdf2-/-
b
*
200
150
100
50
0
Diameter (μm)
Wild type
Ythdf2-/-
c
D3
D5
S100-β/Tuj1/DAPI
S100-β/Tuj1/DAPI
Wild type
Ythdf2-/-
d
80
60
40
20
0
Immunopositive cells (%)
Wild type
Ythdf2-/-
***
**
D3
D5
D3
D5
Tuj1
S100-β
e
TUNEL
TUNEL/DAPI
Wild type
Ythdf2-/-
f
**
60
50
40
30
20
10
0
TUNEL-positive cells (%)
Wild type
Ythdf2-/-

Fig S3.

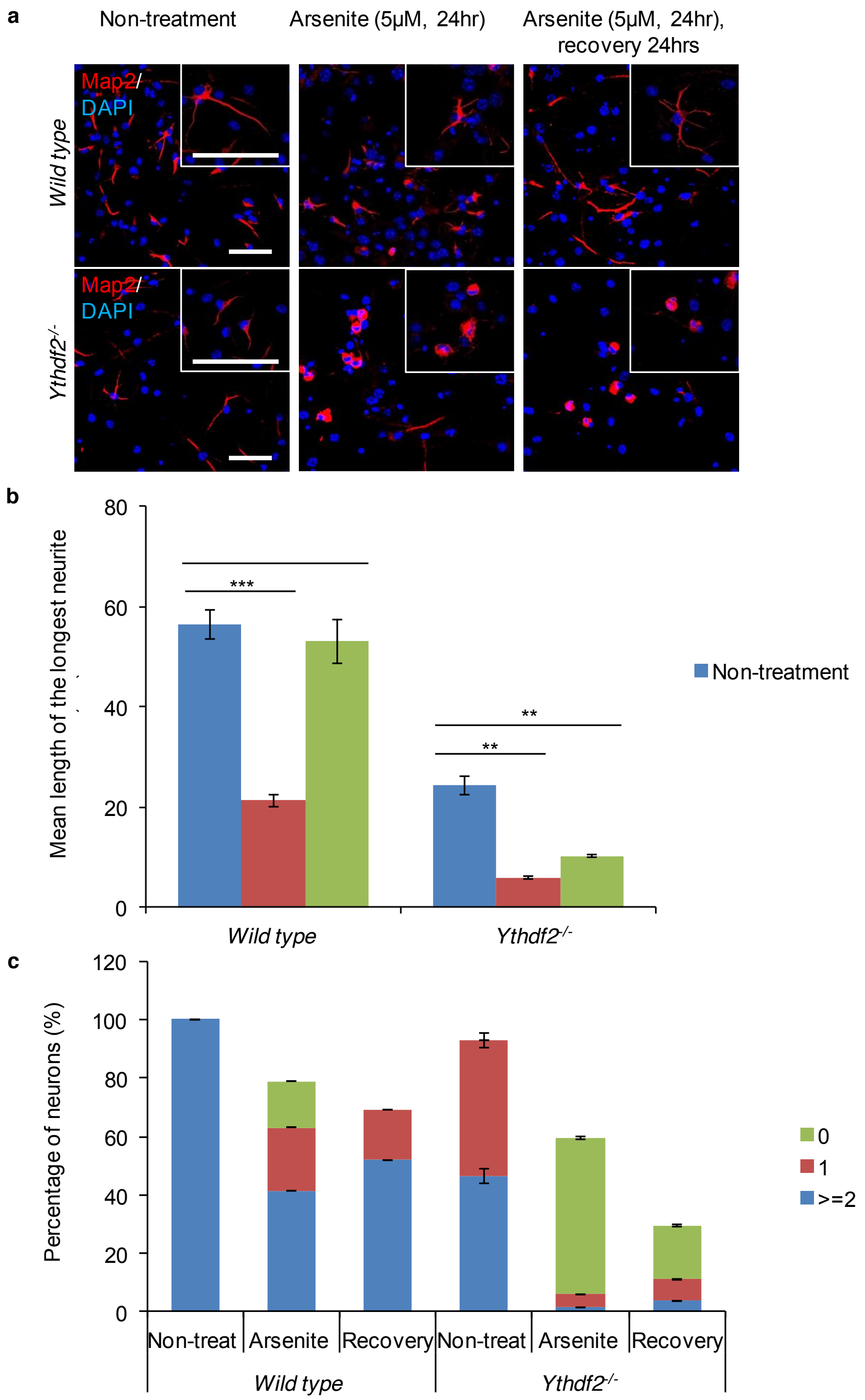

a
Non-treatment
Arsenite (5μM, 24hr)
Arsenite (5μM, 24hr), recovery 24hrs
Map2/
DAPI
Wild type
Ythdf2-/-
b
Mean length of the longest neurite
80
60
40
20
0
***
**
**
Non-treatment
Wild type
Ythdf2-/-
c
Percentage of neurons (%)
120
100
80
60
40
20
0
0
1
>=2
Non-treat
Arsenite
Recovery
Non-treat
Arsenite
Recovery
Wild type
Ythdf2-/-

Fig S4.

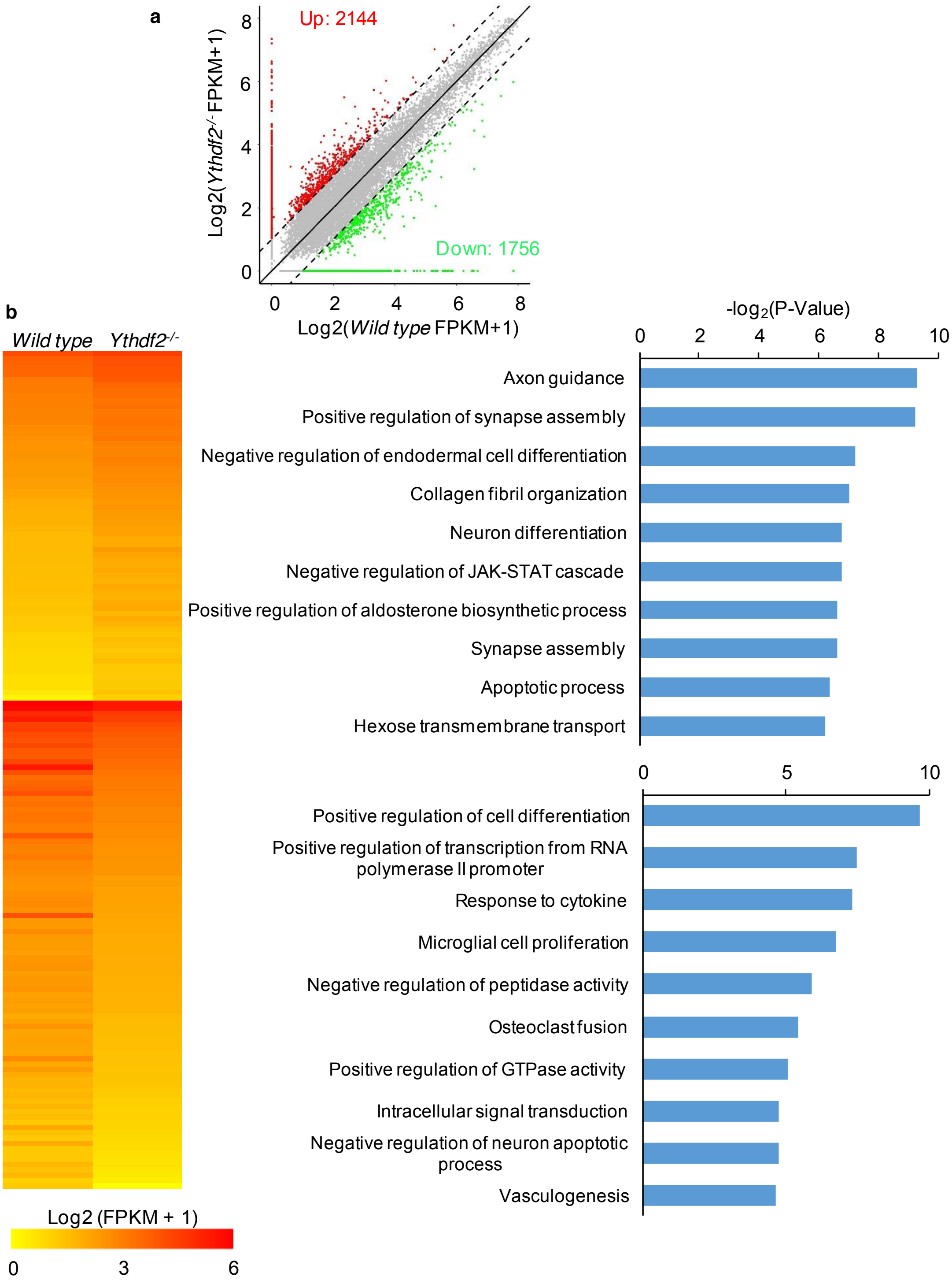

a
Up: 2144
Down: 1756
Log2(Ythdf2-/- FPKM+1)
Log2(Wild type FPKM+1)
0
2
4
6
8
b
Wild type
Ythdf2-/-
Log2 (FPKM + 1)
0
3
6
-log2(P-Value)
Axon guidance
Positive regulation of synapse assembly
Negative regulation of endodermal cell differentiation
Collagen fibril organization
Neuron differentiation
Negative regulation of JAK-STAT cascade
Positive regulation of aldosterone biosynthetic process
Synapse assembly
Apoptotic process
Hexose transmembrane transport
Positive regulation of cell differentiation
Positive regulation of transcription from RNA polymerase II promoter
Response to cytokine
Microglial cell proliferation
Negative regulation of peptidase activity
Osteoclast fusion
Positive regulation of GTPase activity
Intracellular signal transduction
Negative regulation of neuron apoptotic process
Vasculogenesis

Fig S5.

a

	Sample1				Sample2			
**Samep1 vs Sampe2**	**Total**	**Unique**	**Common**		**Common**	**Unique**	**Total**	
KO.R1 vs R2	23301	3713	19588	84.1%	19706	3158	22864	86.2%
KO.R1.R2.common vs R3	19588	1854	17734	90.5%	17935	4590	22525	79.6%
WT vs KO	16626	1576	15050	90.5%	14889	2845	17734	84.0%
WT.R1.R2.common vs R3	19467	2841	16626	85.4%	16764	5294	22058	76.0%
WT.R1 vs R2	22635	3168	19467	86.0%	19244	4016	23260	82.7%

b

Wild type

Rank	Motif	P-value	log P-pvalue	% of Targets	% of Background
1	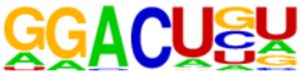	1e-471	-1.085e+03	60.18%	42.23%
2	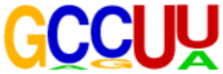	1e-96	-2.224e+02	54.17%	46.08%
3	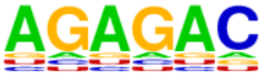	1e-36	-8.379e+01	12.43%	9.43%

$Ythdf2^{-/-}$

Rank	Motif	P-value	log P-pvalue	% of Targets	% of Background
1	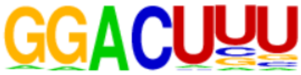	1e-475	-1.094e+03	56.73%	39.35%
2	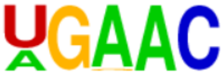	1e-215	-4.955e+02	79.00%	68.46%
3	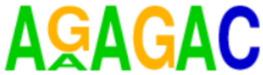	1e-59	-1.374e+02	21.48%	16.74%

Fig S6.

a

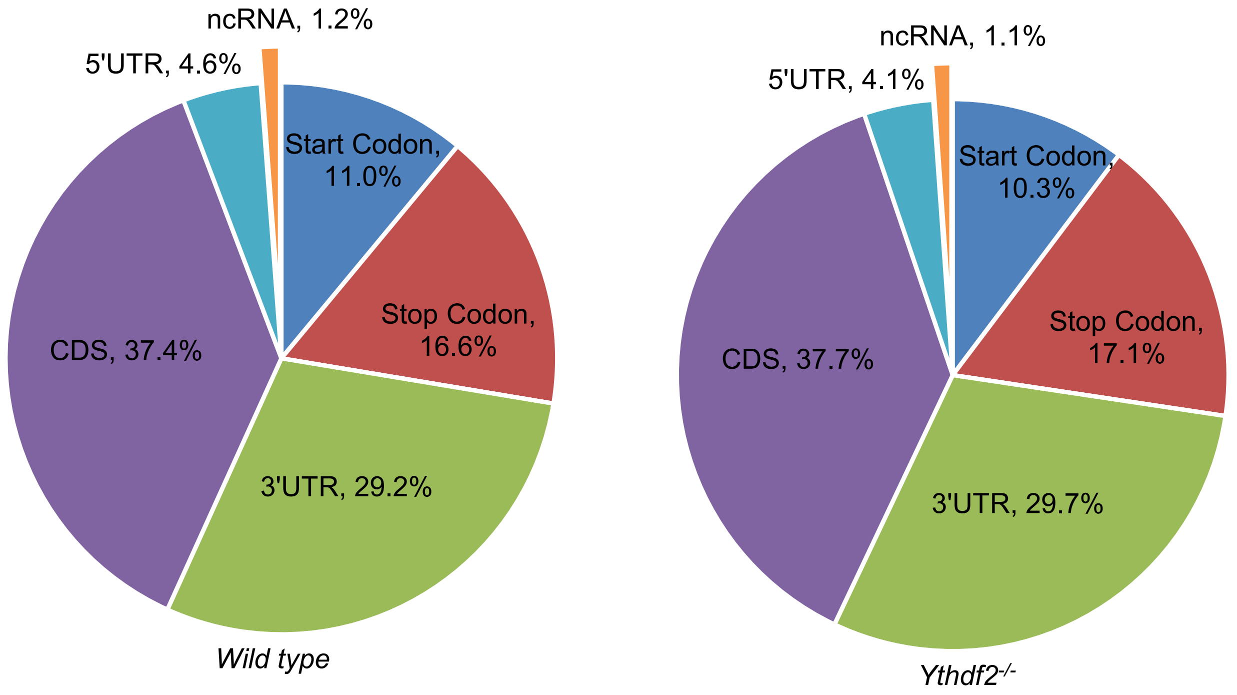

ncRNA, 1.2%
5'UTR, 4.6%
Start Codon, 11.0%
Stop Codon, 16.6%
CDS, 37.4%
3'UTR, 29.2%
Wild type
ncRNA, 1.1%
5'UTR, 4.1%
Start Codon, 10.3%
Stop Codon, 17.1%
CDS, 37.7%
3'UTR, 29.7%
Ythdf2$^{-/-}$

b

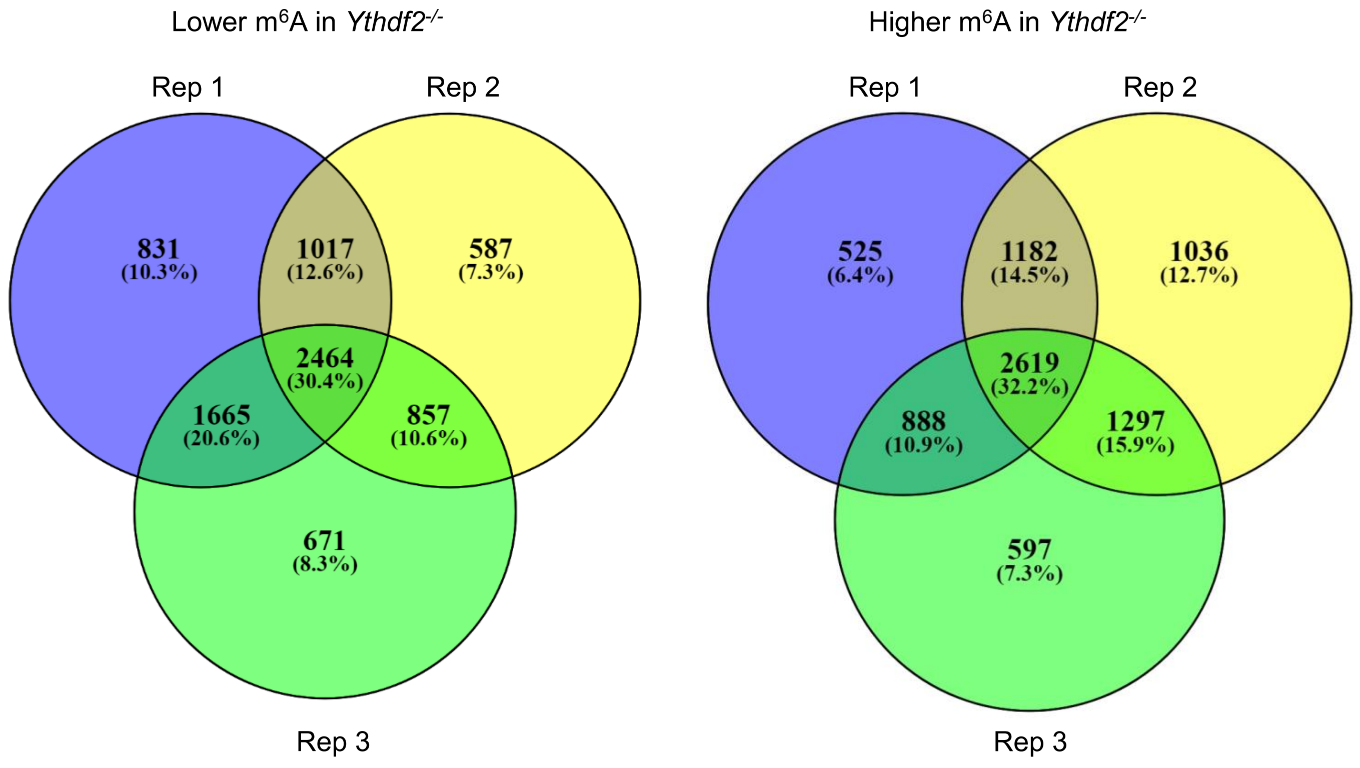

Lower m^{6}A in *Ythdf2*$^{-/-}$
Rep 1
Rep 2
831 (10.3%)
1017 (12.6%)
587 (7.3%)
2464 (30.4%)
1665 (20.6%)
857 (10.6%)
671 (8.3%)
Rep 3
Higher m^{6}A in *Ythdf2*$^{-/-}$
Rep 1
Rep 2
525 (6.4%)
1182 (14.5%)
1036 (12.7%)
2619 (32.2%)
888 (10.9%)
1297 (15.9%)
597 (7.3%)
Rep 3

Fig S7.

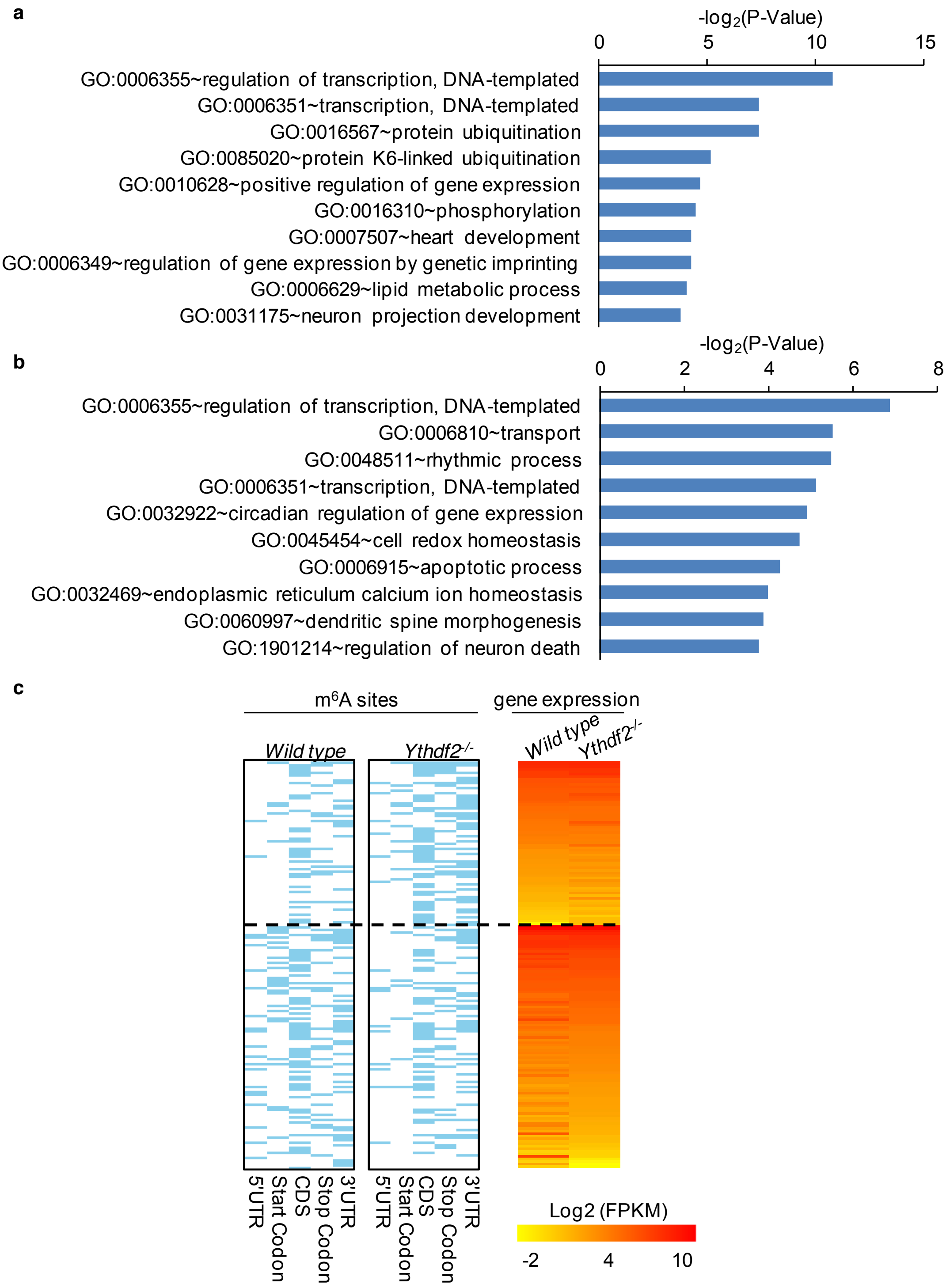
a
-log$_2$(P-Value)
0
5
10
15
GO:0006355~regulation of transcription, DNA-templated
GO:0006351~transcription, DNA-templated
GO:0016567~protein ubiquitination
GO:0085020~protein K6-linked ubiquitination
GO:0010628~positive regulation of gene expression
GO:0016310~phosphorylation
GO:0007507~heart development
GO:0006349~regulation of gene expression by genetic imprinting
GO:0006629~lipid metabolic process
GO:0031175~neuron projection development
b
-log$_2$(P-Value)
0
2
4
6
8
GO:0006355~regulation of transcription, DNA-templated
GO:0006810~transport
GO:0048511~rhythmic process
GO:0006351~transcription, DNA-templated
GO:0032922~circadian regulation of gene expression
GO:0045454~cell redox homeostasis
GO:0006915~apoptotic process
GO:0032469~endoplasmic reticulum calcium ion homeostasis
GO:0060997~dendritic spine morphogenesis
GO:1901214~regulation of neuron death
c
m^{6}A sites
gene expression
Wild type
Ythdf2$^{-/-}$
Wild type
Ythdf2$^{-/-}$
5'UTR
Start Codon
CDS
Stop Codon
3'UTR
5'UTR
Start Codon
CDS
Stop Codon
3'UTR
Log2 (FPKM)
-2
4
10

Fig S8.

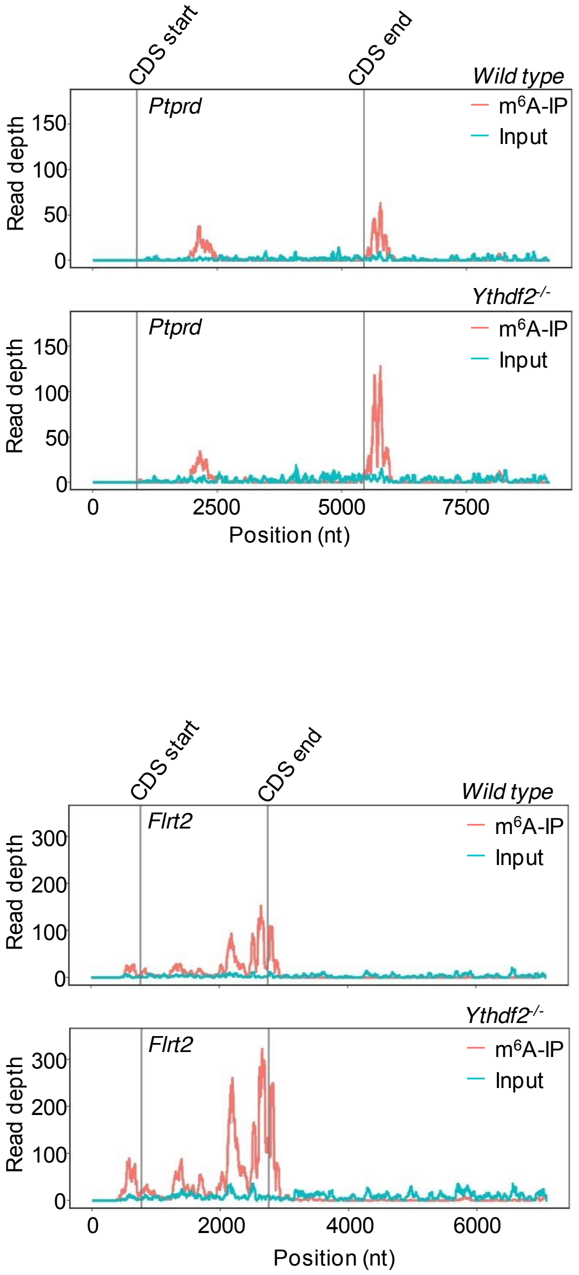

CDS start
CDS end
Wild type
Ptprd
m6A-IP
Input
Read depth
150
100
50
0
Ythdf2-/-
Ptprd
m6A-IP
Input
Read depth
150
100
50
0
0
2500
5000
7500
Position (nt)
CDS start
CDS end
Wild type
Flrt2
m6A-IP
Input
Read depth
300
200
100
0
Ythdf2-/-
Flrt2
m6A-IP
Input
Read depth
300
200
100
0
0
2000
4000
6000
Position (nt)

Fig S9.

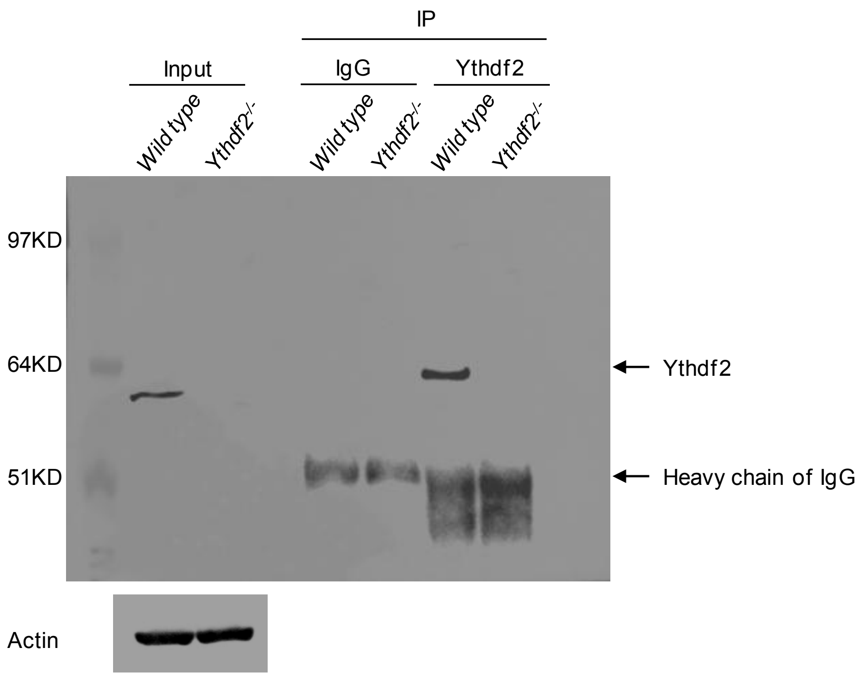

IP
Input
IgG
Ythdf2
Wild type
Ythdf2-/-
Wild type
Ythdf2-/-
Wild type
Ythdf2-/-
97KD
64KD
51KD
Ythdf2
Heavy chain of IgG
Actin

Fig S10.

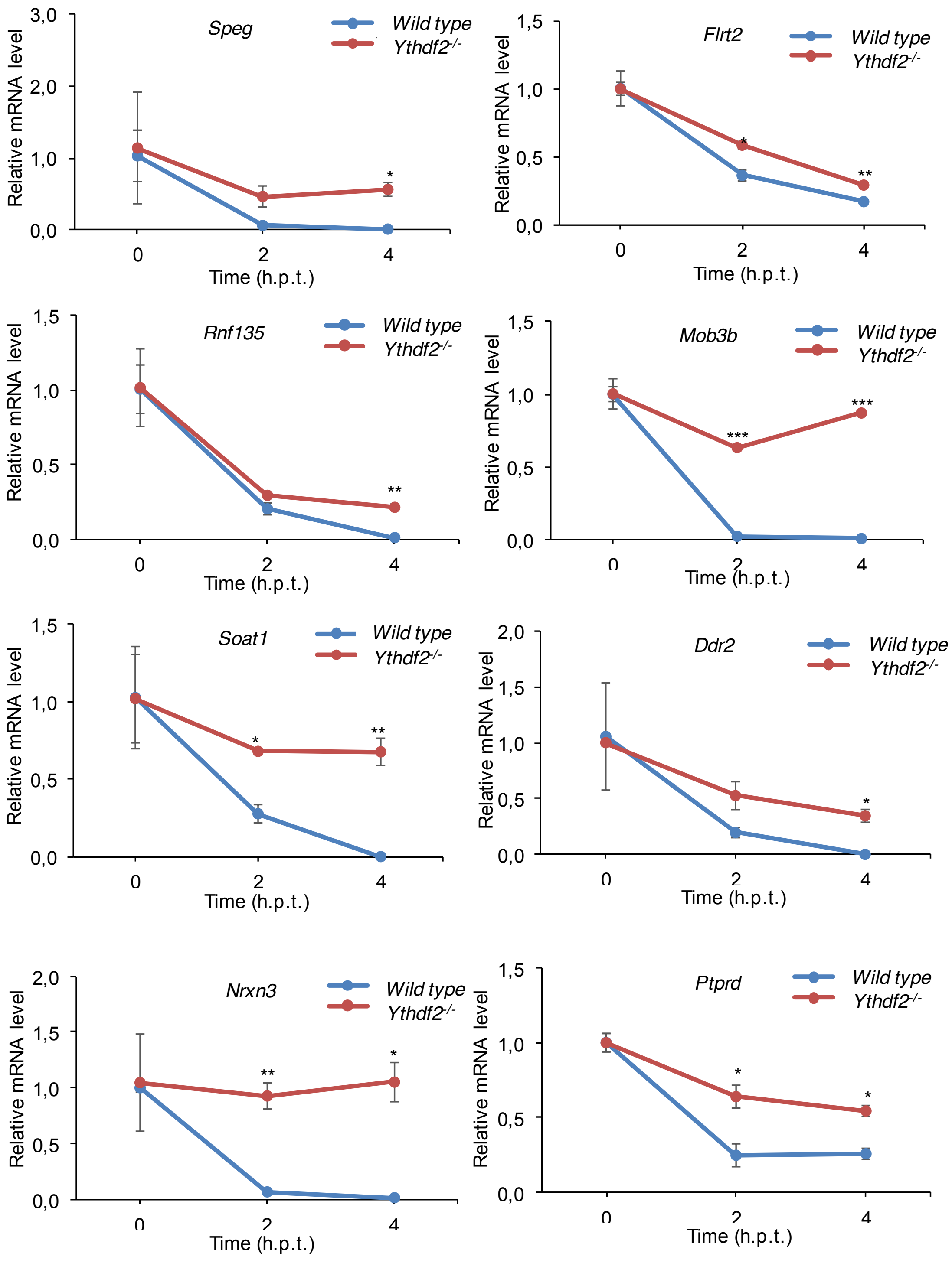
Speg
Wild type
Ythdf2-/-
Relative mRNA level
Time (h.p.t.)
Flrt2
Rnf135
Mob3b
Soat1
Ddr2
Nrxn3
Ptprd
